# Measures of fidelity of delivery of, and engagement with, complex, face‐to‐face health behaviour change interventions: A systematic review of measure quality

**DOI:** 10.1111/bjhp.12260

**Published:** 2017-08-01

**Authors:** Holly Walton, Aimee Spector, Ildiko Tombor, Susan Michie

**Affiliations:** ^1^ Department of Clinical, Educational and Health Psychology University College London UK; ^2^ Department of Epidemiology and Public Health University College London UK

**Keywords:** fidelity of delivery, engagement, measures, quality, psychometric, implementation, complex intervention, health, behaviour change

## Abstract

**Purpose:**

Understanding the effectiveness of complex, face‐to‐face health behaviour change interventions requires high‐quality measures to assess fidelity of delivery and engagement. This systematic review aimed to (1) identify the types of measures used to monitor fidelity of delivery of, and engagement with, complex, face‐to‐face health behaviour change interventions and (2) describe the reporting of psychometric and implementation qualities.

**Methods:**

Electronic databases were searched, systematic reviews and reference lists were hand‐searched, and 21 experts were contacted to identify articles. Studies that quantitatively measured fidelity of delivery of, and/or engagement with, a complex, face‐to‐face health behaviour change intervention for adults were included. Data on interventions, measures, and psychometric and implementation qualities were extracted and synthesized using narrative analysis.

**Results:**

Sixty‐six studies were included: 24 measured both fidelity of delivery and engagement, 20 measured fidelity of delivery, and 22 measured engagement. Measures of fidelity of delivery included observation (*n* = 17; 38.6%), self‐report (*n* = 15; 34%), quantitatively rated qualitative interviews (*n* = 1; 2.3%), or multiple measures (*n* = 11; 25%). Measures of engagement included self‐report (*n* = 18; 39.1%), intervention records (*n* = 11; 24%), or multiple measures (*n* = 17; 37%). Fifty‐one studies (77%) reported at least one psychometric or implementation quality; 49 studies (74.2%) reported at least one psychometric quality, and 17 studies (25.8%) reported at least one implementation quality.

**Conclusion:**

Fewer than half of the reviewed studies measured both fidelity of delivery of, and engagement with complex, face‐to‐face health behaviour change interventions. More studies reported psychometric qualities than implementation qualities. Interpretation of intervention outcomes from fidelity of delivery and engagement measurements may be limited due to a lack of reporting of psychometric and implementation qualities.

**Statement of contribution**

***What is already known on this subject?***

- Evidence of fidelity and engagement is needed to understand effectiveness of complex interventions
- Evidence of fidelity and engagement are rarely reported
- High‐quality measures are needed to measure fidelity and engagement

***What does this study add?***

- Evidence that indicators of quality of measures are reported in some studies
- Evidence that psychometric qualities are reported more frequently than implementation qualities
- A recommendation for intervention evaluations to report indicators of quality of fidelity and engagement measures

## Background

Most interventions aimed at changing health behaviours are complex in that they contain multiple components (Campbell *et al*., 2000; Oakley *et al*., 2006). The effectiveness of face‐to‐face interventions depends on providers delivering the intervention as intended and participants engaging with the intervention. However, delivering interventions with fidelity of delivery and ensuring that participants engage with interventions are not easy to achieve (Glasziou *et al*., 2010; Hardeman *et al*., 2008; Lorencatto, West, Bruguera, & Michie, 2014; Michie *et al*., 2008). Furthermore, it is more difficult to ensure that complex interventions are delivered as intended and engaged with, than simple interventions (Dusenbury & Hansen, 2004; Greenhalgh *et al*., 2004).

To understand, and potentially improve, intervention effectiveness, it is necessary to measure the extent to which the intervention is delivered in line with the protocol (‘intervention fidelity’) and engaged with by participants. Although many conceptualizations of engagement have been proposed (Angell, Matthews, Barrenger, Watson, & Draine, 2014), in this review, the term ‘participant engagement’ is used as an umbrella term to encapsulate constructs of fidelity that relate to participants’ engagement with intervention content. This includes whether participants understand the intervention, whether they can perform the skills required by the intervention (‘intervention receipt’), and whether they use these skills in daily life (‘intervention enactment’) (Borrelli, 2011). In doing this, the review makes a clear distinction between providers’ behaviours (fidelity of delivery) and participants’ behaviours (engagement). Both fidelity of delivery and engagement are necessary to understand the effects of the intervention; if effects are not found, this may be due to low fidelity of delivery and/or engagement and is therefore not a test of the potential of the intervention components (‘active ingredients’) to bring about change (Borrelli, 2011; Durlak, 1998; Lichstein, Riedel, & Grieve, 1994).

Fidelity of delivery has been assessed by self‐report measures (Bellg *et al*., 2004), and by audio‐recording, which is considered to be the gold standard (Bellg *et al*., 2004; Borrelli, 2011; Lorencatto *et al*., 2014). Methods used to assess engagement include self‐report measures (Bellg *et al*., 2004; Burgio *et al*., 2001; Carroll *et al*., 2007), observation of skills (Burgio *et al*., 2001), and homework reviews (Bellg *et al*., 2004). Systematic reviews of measures used to monitor fidelity of delivery demonstrate that these measures have consistently been used in intervention research, in both educational (Maynard, Peters, Vaughn, & Sarteschi, 2013) and health settings (Rixon *et al*., 2016). For example, a review of 55 studies found that intervention receipt was mostly measured by assessing understanding and performance of skills (Rixon *et al*., 2016). Observational measures may provide a more valid representation of what is delivered than self‐report measures (Breitenstein *et al*., 2010) and avoid social desirability bias (Schinckus, Van den Broucke, Housiaux, & Consortium, 2014). However, observation is likely to require more time and resources (Breitenstein *et al*., 2010; Schinckus *et al*., 2014), and it may also change the behaviour of those being observed (McMahon, 1987; as cited in Moncher & Prinz, 1991).

To understand which components have been delivered and engaged with, suitable measures are needed. Researchers suggest that measures should be psychometrically robust, with good reliability and validity (Gearing *et al*., 2011; Glasgow *et al*., 2005; Lohr, 2002; Stufflebeam, 2000). Reliability is defined as achieving consistent results in different situations (Roberts, Priest, & Traynor, 2006), and validity is defined as measurement of the construct it aims to measure (Roberts *et al*., 2006). Previous reviews found that few studies reported information on the reliability or validity of fidelity or engagement methods. A systematic review of fidelity of delivery in after‐school programmes found that no studies reported reliability (Maynard *et al*., 2013), and a systematic review of intervention receipt in health research found that 26% of studies reported on reliability and validity (Rixon *et al*., 2016). This makes it difficult for researchers to fully interpret the quality of measures and therefore the results of intervention outcomes. In this review, we use the term ‘psychometric qualities’ to refer to the quality of the measures. Aspects of ‘psychometric qualities’ of measures in the fidelity literature include the following: using multiple, independent researchers to rate fidelity of delivery; calculating inter‐rater agreement of measurements; and randomly selecting data (Bellg *et al*., 2004; Borrelli, 2011; Breitenstein *et al*., 2010; Lorencatto, West, Seymour, & Michie, 2013).

It is also necessary to ensure that measures are easy to use in practice and to minimize missing responses, which are common in health care self‐report research (Shrive, Stuart, Quan, & Ghali, 2006). Researchers suggest that practicality and acceptability influence the extent to which measures are used in practice (Glasgow *et al*., 2005; Holmbeck & Devine, 2009; Lohr, 2002). Practicality is defined as whether the measure can be used despite limited resources (Bowen *et al*., 2009), for example, being short and easy to use, and reducing participant and provider burden (Glasgow *et al*., 2005; Lohr, 2002). Acceptability is defined as whether the measure is appropriate for those who will use it (Bowen *et al*., 2009), for example, by including alternative forms and language adaptations, and by ensuring that measures are easy to interpret (Lohr, 2002). In this review, we use the term ‘implementation qualities’ to refer to descriptions of how the measures were implemented in practice. Aspects of ‘implementation qualities’ of measures in the fidelity literature include time constraints, cost, and reactions to measurements (Breitenstein *et al*., 2010).

Previous reviews have identified the measures used to monitor fidelity of delivery of after‐school programmes (Maynard *et al*., 2013), evidence‐informed interventions (Slaughter, Hill, & Snelgrove‐Clarke, 2015), and the measures used to monitor intervention receipt in health care settings (Rixon *et al*., 2016). Furthermore, researchers have previously outlined some strengths and weaknesses of different measures of fidelity of delivery and engagement (e.g., Borrelli, 2011; Breitenstein *et al*., 2010; Moncher & Prinz, 1991). To the authors’ knowledge, no systematic reviews have been conducted to identify the measures used to monitor fidelity of delivery and engagement (including intervention receipt and enactment), in complex, face‐to‐face health behaviour change interventions. This review will also extend previous research by describing the reporting of both psychometric and implementation qualities of these measures. Synthesizing the psychometric and implementation qualities of fidelity of delivery and engagement measures is needed to determine the quality of measures and how easy they are to implement. ‘Health’ includes physical, mental, and social well‐being, as recommended by the World Health Organisation (WHO, 2017).

This review aimed to:

1. Identify the types of measures used to monitor (1) the fidelity of delivery of, and (2) engagement with, complex, face‐to‐face health behaviour change interventions.
2. Describe these measures as reported in terms of both psychometric and implementation qualities.

## Methods

The search and screening strategies were developed using the methods advocated by the Cochrane Collaboration (Higgins & Green, 2011; Lefebvre, Manheimer, & Glanville, 2011). Eligibility criteria for considering studies were specified using the ‘Participants’, ‘Intervention’, and ‘Outcomes’ criteria from PICO (O'Connor, Green, & Higgins, 2011).

### Inclusion criteria

1. Participants: Adults aged 18 and over.
2. Intervention: Complex, face‐to‐face behaviour change interventions aimed at improving health behaviours. Health is defined as physical, mental, or social well‐being (WHO, 1946; as cited in WHO, 2017). Other modes of intervention delivery, such as digital interventions, may have different issues in relation to fidelity of delivery and engagement; therefore, these were not included in this review.
3. Outcomes: Studies which described measures to monitor fidelity of delivery and/or engagement and reported outcomes for fidelity of delivery and/or engagement and intervention effectiveness using quantitative measures. Only quantitative studies were included to increase the ability to compare across studies.

### Exclusion criteria

1. Review articles, articles not written in English, or articles not peer‐reviewed
2. Articles in which the intervention outcome could not be clearly distinguished from the engagement or fidelity of delivery outcome.

### Search strategy

Five electronic databases (PubMed, ScienceDirect, PsycINFO, Embase, and CINAHL Plus) were searched from the inception of each database up to November 2015. *Implementation Science* was searched, and reference lists of relevant known reviews (Carroll *et al*., 2007; Durlak & DuPre, 2008; Toomey, Currie‐Murphy, Matthews, & Hurley, 2015) were screened to identify additional studies. After the initial search, reference lists of reviews identified from the search (Clement, Ibrahim, Crichton, Wolf, & Rowlands, 2009; Conn, Hafdahl, Brown, & Brown, 2008; Gucciardi, Chan, Manuel, & Sidani, 2013; Reynolds *et al*., 2014; Smith, Soubhi, Fortin, Hudon, & O'Dowd, 2012), relevant protocols (Gardner *et al*., 2014), and forward and backward searching of included studies were screened to identify further articles. The articles generated by this search strategy were sent to 21 experts to ask whether they knew of relevant articles that were missing from the search results.

Initial search terms were piloted and refined iteratively with sequential testing to identify false‐positive and false‐negative results and ensure that the search captured all relevant keywords. A subject librarian was consulted in the development of the search terms.

Free and mapped searches (using Medical Subject Heading Terms) were conducted. Boolean operators were used to construct a search incorporating all search terms when combination searches were not possible. Search outputs were filtered for English full texts, peer‐reviewed articles, adult participants and health topics. The final search strategy is in Appendix S1.

To access articles not available through the university library database, the authors were contacted or articles were accessed through library services.

This search strategy was not exhaustive, but was instead used to identify as many papers that measured and reported fidelity of delivery and/or engagement in sufficient depth to provide insight into the measures used.

### Data collection and analysis

#### Study selection

One reviewer conducted the electronic searches and screened the reference lists of relevant articles. All identified titles and abstracts were downloaded and merged using EndNote. Duplicates were removed. Two reviewers independently screened all (1) titles, (2) abstracts, and (3) full texts against inclusion and exclusion criteria. Reviewers met after each stage to determine agreement and resolve discrepancies. Any articles which reviewers were unsure of were retained until data extraction, when more information was available (Higgins & Deeks, 2008). Inter‐rater reliability was assessed using percentage agreement and kappa statistics. Scores from both the initial search screening and additional search screening were combined to calculate agreement scores. For the title screening, researchers achieved 64.9% agreement (*n* = 802, two missing responses, kappa .49, PABAK .47). For the abstract screening, researchers achieved 68% agreement (*n* = 425, three missing responses, kappa .36, PABAK .36). For the full‐text screening, researchers achieved 71.8% agreement (*n* = 266; kappa = .46 and PABAK = .58). The full‐text kappa scores (Cohen, 1960) indicated fair agreement (Orwin, 1994; as cited in Higgins & Deeks, 2008). This might reflect the difficulty identifying relevant articles due to differences in terminology in studies. Information on fidelity of delivery and engagement was often reported in separate articles than those reporting intervention outcomes.

#### Data extraction

A data extraction form was developed using a combination of standardized forms: Guidelines International Network‐Evidence Tables Working Group intervention template (Guidelines International Network, 2002–2017) and the Oxford Implementation Index (Montgomery, Underhill, Gardner, Operario, & Mayo‐Wilson, 2013). Data on the measures used to monitor fidelity of delivery and engagement and results were extracted, along with any qualities of measures that were reported. Psychometric qualities and implementation qualities were not pre‐specified before data extraction; therefore, any information that was reported in the results and discussion section of the original articles in relation to the quality of the measures was extracted. As a minimum quality check (Centre for Reviews and Dissemination, University of York, 2009), an independent researcher checked 20% of data extraction forms. Minor errors of punctuation were identified; however, no further details were extracted, and therefore, one researcher extracted data from all studies.

#### Data synthesis

Narrative analysis was used to summarize the fidelity of delivery and engagement measures and the reporting of psychometric and implementation qualities by one researcher. If authors specified the type of engagement that they measured, for example, ‘intervention receipt’ or ‘intervention enactment’, these were reported separately within engagement. One researcher synthesized the information on methods. The extracts from the text that included descriptions of qualities were summarized, and the part of the procedure that the quality related to was recorded. Psychometric qualities included reliability (achieving consistent results in different situations; Roberts *et al*., 2006) and validity (measures what it aims to measure; Roberts *et al*., 2006). Implementation qualities included acceptability (appropriate for those who will use it; Bowen *et al*., 2009), practicality (can be used despite limited resources; Bowen *et al*., 2009), and cost. Researchers were open to other categories that may have emerged if qualities did not fit into these categories. Due to the heterogeneity of studies, a descriptive rather than quantitative synthesis of data was conducted (Deeks, Higgins, & Altman, 2008; Popay *et al*., 2006).

Two researchers were involved in the categorization of psychometric and implementation qualities. The first author coded 10% of the qualities and asked an independent researcher to check responses. Disagreements were identified, and both researchers independently coded an additional 10% of qualities. Researchers met after each round to discuss disagreements. This process was repeated, until 80% agreement on the categorization of features was reached, as recommended by Lombard, Snyder‐Duch, and Bracken (2002). After four rounds (40% of qualities were independently coded), reliability was achieved with 80.1% agreement between coders. The first author coded the rest of the qualities, based on discussions with the second researcher. Following this, the second researcher checked a further 10% of the researcher's independent coding and any qualities that the first author was unsure how to code.

## Results

**Figure 1:**
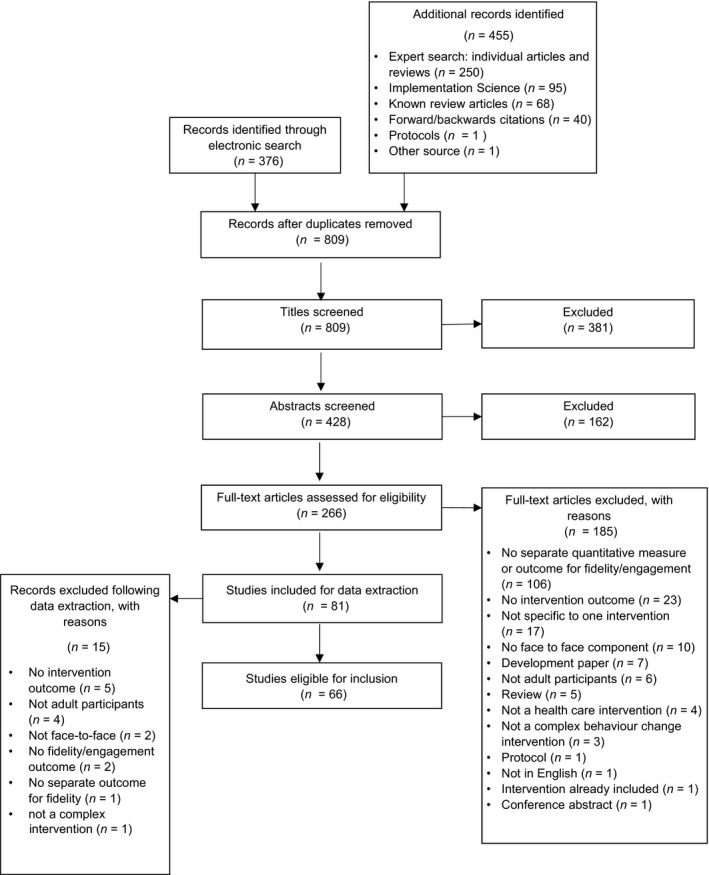
A flow diagram of the paper selection process (based on Moher, Liberati, Tetzlaff, and Altman's (2009) PRISMA flow diagram).

After duplicates were removed, 809 records were identified. Sixty‐six articles were included in the analysis (Figure 1).

### Study characteristics

Sixty‐six studies (100%) were included (for a list of studies and their characteristics, see Appendix S2). All of the included studies described fidelity of delivery and/or engagement measures, in relation to a complex, face‐to‐face health behaviour change intervention. Forty‐six studies (69.7%) were randomized controlled trials and 20 (30.3%) used non‐randomized designs. Settings included medical settings (*n* = 40; 60.6%), community settings (*n* = 20; 30.3%), and companies (*n* = 1; 1.5%). Five studies (7.6%) did not specify their setting. Intervention recipients were patients (*n* = 31; 47%), members of the public (*n* = 17; 25.8%), health care professionals and practices (*n* = 11; 16.7%), caregivers and care recipients (*n* = 4; 6.1%), and workers (*n* = 3; 4.5%). Target behaviours included multiple health behaviours (*n* = 35; 53%), self‐management skills (*n* = 11; 16.7%), clinician behaviours (*n* = 10; 15.2%), anxiety‐reducing behaviours (*n* = 3; 4.5%), work sickness absence (*n* = 2; 3%), caregiver skills (*n* = 2; 3%), treatment adherence (*n* = 1; 1.5%), patient resource use (*n* = 1; 1,5%), and activities of daily living (*n* = 1; 1.5%). Interventions were delivered by health care professionals (*n* = 33; 50%), people trained especially for the intervention (e.g., community mediators and outreach visitors) (*n* = 11; 16.7%), pharmacists (*n* = 2; 3%), postgraduate students (*n* = 2; 3%), and researchers (*n* = 4; 6%). Fourteen studies (21.2%) did not specify who delivered the intervention.

### Measures used to monitor fidelity of delivery and engagement

Of all included studies, 44 (66.7%) assessed fidelity of delivery and 46 (69.7%) assessed engagement. Of these, 24 studies (36.4%) measured both fidelity of delivery and engagement, 20 (30.3%) measured fidelity of delivery only, and 22 (33.3%) measured engagement only (see Appendix S3).

Table 1 provides an overview of the methods, including a summary of what was measured, the measures used, who completed the measures, the sample, analysis method, and the number of studies that used a framework/model and provided definitions for fidelity and engagement. For further details about methods and a summary of results, please see Appendix S4.

**Table 1:** A summary of the measures used to monitor fidelity of delivery and engagement

	Fidelity (*n* = 44; 100%)	Engagement (*n* = 46, 100%)
What was measured?	Delivery of intervention components compared with intervention protocol (*n* = 20; 45.5%)^1,5,6,10,11,16,20 (specifically BCTs) 26,28,29,30,31,35,39,40,51,55,59,60,66^ Motivational interviewing adherence/fidelity/infidelity (*n* = 6; 13.6%)^7,22,57,58,63,64^ Dose delivered and fidelity (*n* = 6; 13.6%)^2,14,23,36,42,49^ Fidelity of delivery but unclear which aspect as results not reported (*n* = 2; 4.5%)^19,21^ Dose of intervention components (*n* = 2; 4.5%)^24,62^ Competence and success delivering behaviour change strategies (*n* = 1; 2.3%)^41^ Treatment integrity/demonstration of skills (*n* = 1; 2.3%)^25^ Extent to which environmental changes made (*n* = 1; 2.3%)^50^ Consistency and quality of use of innovation (*n* = 1; 2.3%)^33^ Motivational interviewing fidelity, dose, and context (*n* = 1; 2.3%)^38^ ‘Quality of counselling’ – use of skills and therapeutic alliance (*n* = 1; 2.3%)^27^ Number of times skills were modelled and telephone fidelity (*n* = 1; 2.3%)^34^ Clinician competence/demonstration of intervention method (*n* = 1; 2.3%)^48^	Adherence to target behaviour (*n* = 7; 15.2%)^3,4(+Skills),13,15,19,37,43^ Attendance (*n* = 7; 15.2%)^9,40,44,46,54,56,65^ Understanding (receipt) and use of intervention skills (enactment) (*n* = 3; 6.5%)^6,35,48^ Understanding and engagement (*n* = 2; 4.34%)^42,51^ Compliance and attendance (*n* = 2; 4.34%)^18,47^ Adherence to target behaviour and attendance (*n* = 2; 4.34%)^17,52^ Completion of study visits (*n* = 2; 4.34%)^21,41,^ Intervention enactment – use of BCTs (*n* = 1; 2.17%)^25^ Receipt, enactment, homework compliance, and attendance (*n* = 1; 2.17%)^39^ Dose received/exposure – assignments completed (*n* = 1; 2.17%)^2^ Dose received – intervention receipt and compliance (*n* = 1; 2.17%)^14^ How much learned/adopted, helpfulness, and current use (*n* = 1; 2.17%)^11^ Effectiveness of intervention – trying practices, participating, influencing practice, comprehension, future participation (*n* = 1; 2.17%)^16^ Adoption of intervention and maintenance (*n* = 1; 2.17%)^29^ Dose of intervention received (*n* = 1; 2.17%)^36^ Receipt and reaching goals (*n* = 1; 2.17%)^30^ Participation in activities, dose, and checklist completion (*n* = 1; 2.17%)^5^ Activity adherence, sessions delivered, telephone contact (*n* = 1; 2.17%)^12^ Adherence to target behaviour and diary (*n* = 1; 2.17%)^38^ Adherence to target behaviour, attendance, and diary (*n* = 1; 2.17%)^53^ Exposure to intervention – attendance/receipt of calls (*n* = 1; 2.17%)^32^ Uptake of intervention – attendance/use of modules (*n* = 1; 2.17%)^8^ Attendance, reading materials, usefulness, meeting goals (*n* = 1; 2.17%)^61^ Attendance and completion of diaries (*n* = 1; 2.17%)^64^ Completion of diaries (*n* = 1; 2.17%)^10^ Completion of home assignments, self‐monitoring, attendance (*n* = 1; 2.17%)^23^ Homework adherence and commitment (*n* = 1; 2.17%)^24^ Completion of homework, receipt of information, telephone calls (*n* = 1; 2.17%)^55^
Type of measures used	Observational measures (*n* = 17; 38.6%): Video (*n* = 2; 4.55%)^27,51^ Audio (*n* = 13; 29.5%)^7,19,21,22,38,40,45,48,55,57,58,63,64^ Non‐specific (*n* = 2; 4.55%)^1,34^ Self‐report measures (*n* = 15; 34%): Provider (hand) (*n* = 7; 15.9%)^6,10,14,16,41,42,59^ Provider (computer) (*n* = 3; 6.8%)^24,23,36^ Participant (hand) (*n* = 2; 4.6%)^28,11^ Participant (computer) (*n* = 1; 2.3%)^49^ Non‐specific (computer) (*n* = 2; 4.6%)^62,66^ Multiple measures (*n* = 11; 25%) Provider and participant self‐report (*n* = 4; 9%)^2,30,35,50^ Audio and provider self‐report (*n* = 3; 6.8%)^20,26,39^ Video + provider self‐report (*n* = 1; 2.3%)^5^ Observation and exercise log (participant) (*n* = 1; 2.3%)^31^ Direct observation and rating (*n* = 1; 2.3%)^29^ Participant self‐report and patient files (*n* = 1; 2.3%)^60^ Other measures (*n* = 1; 2.3%) Quantitative rated interviews with providers (*n* = 1; 2.3%)^33^	Self‐report measures (*n* = 18; 39.1%) Participant (*n* = 14; 30.4%)^11,13,14(R),16,19,25,30,35,36,37,38,43,48,55^ Provider (*n* = 4; 8.7%)^10,41,42,51^ Multiple measures (*n* = 17; 37%): Provider and participant self‐report (*n* = 3; 6.5%)^2,3,5^ Participant self‐report and attendance records (*n* = 3; 6.5%)^18,23,32^ Provider and participant self‐report and attendance records (*n* = 2; 4.3%)^17,47^ Attendance records and behaviour monitoring (*n* = 2; 4.3%)^53,64^ Direct observation and provider and participant self‐report (*n* = 1; 2.2%)^12^ Non‐specific observation and provider self‐report (*n* = 1; 2.2%)^4^ Provider self‐report, attendance records, homework review (*n* = 1; 2.2%)^39(R&E)^ Participant self‐report and verbal verification (*n* = 1; 2.2%)^6(R&E)^ Provider self‐report and homework review (*n* = 1; 2.2%)^24^ Participant self‐report and objective verification (*n* = 1; 2.2%)^15^ Provider self‐report and attendance records (*n* = 1; 2.2%)^52^ Intervention records (*n* = 11; 24%) Attendance/referral records (*n* = 10; 21.7%)^8,9,29,40,44,46,54,56,61,65^ Study completion (*n* = 1; 2.2%)^21^
More details about measures	Who completed the measures? Researcher (*n* = 18; 40.9%)^1,7,21,22,27,29,33,34,38,40,45,48,51,55,57,58,63,64^ Provider (*n* = 11; 25%)^6,10,14,16,19,23,24,36,41,42,59^ Provider and participant (*n* = 4; 9.1%)^2,30,35,50^ Provider and researcher (*n* = 4; 9.1%)^5,20,26,39^ Participant (*n* = 3; 6.8%),^11,28,49^ Participant and researcher (*n* = 2; 4.55)^31,60^ Not specified (*n* = 2; 4.55)^62,66^	Who completed the measures? Participant (*n* = 14; 30.4%)^11,13,14(R),16,19,25,30,35,36,37,38,43,48,55^ Researcher (*n* = 13; 28.3%)^8,9,21,29,40,44,46,53,54,56,61,64,65^ Participant and researcher (*n* = 6; 13%)^6(R&E),15,18,23,24,32^ Provider (*n* = 4; 8.7%)^10,41,42,51^ Provider and participant (*n* = 3; 6.5%)^2,3,5^ Provider and researcher (*n* = 3; 6.5%)^4,39(R&E),52^ Provider, participant, researcher (*n* = 3; 6.5%)^12,17,47^
	Development of measures Not specified (*n* = 31; 70.45%)^1,5,11,14,16,19,23,24,27,28,29,30,31,33,35,36,38,39,40,41,42,48,49,50,51,55,59,60,62,64,66^ Used a previously developed measure (*n* = 8; 18.18%) Motivational interviewing treatment integrity code (Moyers *et al*., 2003 as cited in^57,58^, 2007, as cited in^22^): (*n* = 3; 6.8%)^22,57,58^ MITI + Motivational interviewing skill code (Miller *et al*., 2003) (*n* = 2; 4.5%)^7,63^ Behaviour Change Counselling Index (Lane *et al*., 2005) (*n* = 2; 4.5%)^21,45^ Flanders Interaction Analysis Technique (*n* = 1; 2.3%)^34^ Developed own measure: (*n* = 5; 11.36)^2,6,10,20,26^	Development of measures Not specified: (*n* = 42; 91.3%)^2,3,5,6,8,9,10,11,12,13,14,15,16,17,18,19,21,23,24,25,29,30,32,35,36,37,38,39,40,41,42,44,46,47,48,53,54,55,56,61,64,65^ Used previously developed measure (*n* = 3; 6.5%) DASH adherence index: (*n* = 1; 2.17%)^43^ Pittsburgh Rehabilitation Participation scale (*n* = 1; 2.17%)^51 (engagement,understanding not specified)^ Participation scale and the participation scale and recovery practice scale (*n* = 1; 2.17%)^52^ Developed own measure and used measures that were previously developed: (*n* = 1; 2.2%)^4^
	Responses on measures Not specified (*n* = 23; 52.3%)^1,6,7,10,16,19,21,22,23,24,31,34,35,38,39,40,42,48,49,51,62,64,66^ Rating scales (*n* = 12; 27.3%) 3‐point scale (completely covered, partially covered, not covered) (*n* = 1; 2.27%)^5^ 4‐point scale (*n* = 1; 2.27%)^45^ Two 4‐point rating scales (unsatisfactory, doubtful, satisfactory, good’, ‘not at all, hardly, slightly, considerably, strongly’ + Not applicable (*n* = 1; 2.27%)^27^ Two 4‐point scales (‘Excellent, good, fair, poor’ and ‘used well, used well but not often, used well and not well, not used or not used well) (*n* = 1; 2.27%)^29^ 5‐point scale (Totally disagree – totally agree) (*n* = 1; 2.27%)^2^ 5‐point scale (‘Never, most of the time, often, always, do not remember’) (*n* = 1; 2.27%)^30^ 5‐point scale (‘Non‐use, low compliance, compliant use, high compliance, committed use’) (*n* = 1; 2.27%)^33^ 7‐point scale (low (1), high (7)) + behaviour counts (*n* = 2; 4.5%)^57,58^ 7‐point scale (*n* = 1; 2.27%)^63^ Eight point scales (no adherence – optimal adherence and no competence – excellent competency) (*n* = 1; 2.27%)^55^ 10‐point scale (very bad to very good) + three point scale (yes/partly/not implemented) (*n* = 1; 2.27%)^14^ Dichotomous scale: (*n* = 8; 18.2%) Yes/no (*n* = 5; 11.4%)^11,28,41,59,60^ Applied(1)/not applied (0) or completed (1)/not completed (0) (*n* = 2; 4.5%)^20,26^ Completed)(1)/not completed(0) (*n* = 1; 2.27%)^36^ Rating scale and dichotomous scale (*n* = 1; 2.3%) 4‐point scale (rarely (1), sometimes (2), often (3), most/all of the time (4) and yes (1)/no (0) (*n* = 1; 2.3%)^50^	Responses on measures Not specified: (*n* = 29; 63%)^2,3,5,6,8,9,12,13,15,17,18,19,21,23,29,30,32,35,37,38,40,42,44,48,53,54,56,61,65^ Rating scales (*n* = 12; 26.1%) 3‐point scale adherence (poor, fair, excellent), others not specified (*n* = 1; 2.17%)^4^ 3‐point scales: perceived helpfulness (0 not at all, 2 very much) + currently using (0 not at all, 2 very much) (*n* = 1; 2.17%)^11^ 3‐point scale (0 = effectively non‐compliant, 0.5 = uncertain or partly compliant, 1 = compliant) (*n* = 1; 2.17%)^47^ 3‐point scales (yes/no/don't know and ‘very helpful, neither helpful nor unhelpful, very unhelpful’), four point scale (most, all, some, none), (*n* = 1; 2.17%)^36^ 3‐point scale (Better than target range [>1], 0–1 within target range, worse than target range [<0]): (*n* = 1; 2.17%)^43^ 3‐point Likert scale (very low to very high) (*n* = 1; 2.17%)^52^ 3‐point scale (*n* = 1; 2.17%)^64^ 4‐point scale (dissatisfied to very satisfied) (*n* = 1; 2.17%)^55^ 4‐point scale (1 missed most–4 missed none) and 10 point scale (1 none, 10 complete) (*n* = 1; 2.17%)^24^ 5‐point Likert scale: (*n* = 1; 2.17%)^16^ 6‐point Likert scale (1 no engagement, 6 excellent engagement) and 3‐point scale (1 minimal understanding, some understanding, good understanding) (*n* = 1; 2.17%)^51^ 7‐point scale (Never, <3 months ago, 4–6 months ago, 7–9 months ago, 10–12 months ago, 1–2 years ago, <2 years ago) (*n* = 1; 2.17%)^46^ Dichotomous scales (*n* = 3; 6.5%) Yes/no: (*n* = 3; 6.5%)^10,25,41^ Rating scale + dichotomous scale (*n* = 2; 4.4%) 3‐point scale (yes/no/don't know) and dichotomous scale (yes/no): (*n* = 1; 2.17%)^14^ 3‐point scale (0 not at all, fully) – measure receipt. 5‐point scale (1 not at all, 5 extremely) measure willingness, interest and supportiveness and dichotomous scale (attempted, not attempted) – to measure enactment (*n* = 1; 2.17%)^39^
Sample	How many participants were sampled? Not specified (*n* = 23; 52.3%)^1,2,5,7,11,14,16,19,21,22,23,28,34,35,41,42,49,50,57,58,60,62,66^ Subsample (*n* = 16; 36.4%)^10 26,27,29,30,31,33,36,38,40,45,48,51,55,63,64^ Reported number of sessions sampled (*n* = 4; 9%)^26,27,31,63^ Reported number of clinicians/sites data was sampled from (*n* = 4; 9%)^10,29,30,33^ Reported the percentage of sessions sampled (*n* = 6; 13.6%)^36,38,40,45,51,55^ Reported sampling some but not all but did not specify how many (*n* = 2; 4.5%)^48,64^ All (*n* = 5; 11.4%):^6,20,24,39,59^	How many participants were sampled? Not specified (*n* = 45; 97.8%)^2,3,4,5,6,8,9,10,11,12,13,14,15,16,17,18,19,21,23,24,25,29,32,35,36,37,38,39,40,41,42,43,44,46,47,48,51,52,53,54,55,56,61,64,65^ Subsample (*n* = 1; 2.2%)^30^ Reported sampling a number of participants (*n* = 1; 2.2%)^30^
	How were participants sampled? Not specified: (*n* = 25; 56.8%)^1,2,5,7,11,14,16,19,21,22,23,28,29,30,34,35,36,38,41,42,49,50,60,62,66^ Random (*n* = 8; 18.2%)^31,40,51,55,57 (random segment),58 (random segment),63,64,^ N/A (sampled all: *n* = 5; 11.4%)^6,20,24,39,59^ Purposive: (*n* = 3; 6.8%)^26,27 (previously defined days),33^ Self‐selected (*n* = 1; 2.3%)^48^ Opportunity: (*n* = 1; 2.3%)^45^ Stratified: (*n* = 1; 2.3%)^10^	How were participants sampled? Not specified: (*n* = 46; 100%)^2,3,4,5,6,8,9,10,11,12,13,14,15,16,17,18,19,21,23,24,25,29,30,32,35,36,37,38,39,40,41,42,43,44,46,47,48,51,52,53,54,55,56,61,64,65^
	Which conditions were participants sampled from? Not specified (likely intervention only) : (*n* = 38; 86.4%)^1,5,6,10,11,14,16,19,20,21,22,23,26,27,28,29,30,31,33,34,35,36,38,39,40,41,42,45,49,55,57,58,59,60,62,63,64,66^ All: (Explicitly reported) (*n* = 4; 9.1%)^48,51,7,50^ Intervention(s) (*n* = 2; 4.5%)^2,24^	Which conditions were participants sampled from? ☐ Not specified (likely intervention only): (*n* = 35; 76.1%)^5,6,8,9,10,11,12,14,15,16,19,21,23,29,30,32,36,37,38,39,40,41,42,43,44,46,47,48,52,54,55,56,61,64,65^ ☐ All (explicitly reported): (*n* = 9; 19.6%)^2,3,18,35,4,13,17,51,53^ ☐ Intervention(s) (*n* = 2; 4.3%)^24,25^
Analysis method	Descriptive statistics (*n* = 29; 65.9%)^1,5,6,10,11,14,16,22,23,27,28,29,30,31,33,34,36,38,39,41,42,45,49,55,57,58,59,60,66^ Descriptive and inferential statistical techniques (*n* = 11; 25%)^2,7,20,24,26,35,48,50,51 (inferential not specified)^ ^62,63^ Not reported (*n* = 4; 9.1%)^19,21,40,64^	Descriptive statistics (*n* = 37; 80.4%)^3,4,5,6,8,9,10,11,12,14,15,16,18,19,21,23,29,30,32,35,36,37,38,40,41,42,44,46,47,48,52,54,55,56,61,64,65^ Descriptive statistics and Inferential statistical techniques (*n* = 9; 19.6%)^2,13 (inferential stats not specified) 17,24,25,39,43,51,53^
Framework/model	Framework not specified/mentioned (*n* = 53; 80.3%)^1,3,4,5,7,8,9,11 (mentioned in discussion),12,13,15,16,17,18,19,21,23,24,25,27,28,30,32,33,34,35,36,37,38,40,41,43,44,45,46,47,48,49,51,52,53,54,55,56,57,58,59,61,62,63,64,65,66^ Used a framework (*n* = 13; 19.7%)^2,6,10,14,20,22,26,29,31,39,42,50,60^ Steckler and Linnan's (2002, as cited in^2,14,42,50^) framework (*n* = 4; 6.1%)^2,14 (adapted version),42,50^ NIH Treatment fidelity model/NIH Behaviour change Consortium framework (Bellg *et al*., 2004) (*n* = 6; 9.1%)^6,10,20,22,26,39^ RE‐AIM framework (*n* = 1; 1.5%)^29^ Resnick *et al*. (2005) (*n* = 1; 1.5%)^31^ Baranowski & Stables (2000): (*n* = 2; 3.3%)^42,50^ Saunders *et al*. (2005) (*n* = 1; 1.5%)^42^ Hasson (2010) based on Carroll *et al*. (2007) (*n* = 1; 1.5%)^60^	
Definitions	Provided definitions (*n* = 18; 27.3%)^2,5,6,12,14,16,17,20,22,23,25,31,33,38,39,41,42,50^ Fidelity (constructs that fit into fidelity): (*n* = 15; 22.7%)^2,5,6,14,16,20,22,23,31,33,38,39,41,42,50^ Engagement (constructs that fit under engagement): (*n* = 9; 13.6%)^2,6,12,14,17,23,25,39,42^ Did not provide definitions (*n* = 48; 72.7%)^1,3,4,7,8,9,10,11,13,15,18,19,21,24,26,27,28,29,30,32,34,35,36,37,40,43,44,45,46,47,48,49,51,52,53,54,55,56,57,58,59,60,61,62,63,64,65,66^	

Note

(R) = receipt; (E) = enactment; (R&E) = receipt and enactment.

#### What was measured?

The majority of studies reporting measuring fidelity of delivery did so by measuring the delivery of intervention components against the intervention protocol (*n* = 20; 45.5%), adherence to motivational interviewing techniques (*n* = 6; 13.6%), and a combination of dose delivered and fidelity (*n* = 6; 13.6%). For engagement, there were a wide variety of measures, including adherence to target behaviour (*n* = 7; 15.2%), attendance (*n* = 7; 15.2%), understanding and use of intervention skills (*n* = 3; 6.5%), understanding and engagement (*n* = 2; 4.4%), compliance and attendance (*n* = 2; 4.4%), adherence to target behaviour and attendance (*n* = 2; 4.4%), and completion of study visits (*n* = 2; 4.4%). Please see Table 1 for a full list of what was measured.

#### Measures

Measures of fidelity of delivery were categorized into observational measures (*n* = 17; 38.6%), self‐report measures (*n* = 15; 34%), quantitatively rated qualitative interviews (*n* = 1; 2.3%), and multiple measures (*n* = 11; 25%). Of the studies that used multiple measures, six (14%) used at least one type of observational measure and nine (20.5%) used at least one type of self‐report measure. In total, 23 (52%) studies used at least one type of observational measure and 24 (55%) used at least one type of self‐report measure (see Table 1 for details).

Measures of engagement were categorized into self‐report measures (*n* = 18; 39.1%); intervention records (*n* = 11; 24%), for example, attendance monitoring; and multiple measures (*n* = 17, 37%). Of the studies that used multiple measures, 15 (32.6%) used at least one type of self‐report measure. In total, 33 (76.7%) studies used at least one type of self‐report measure (see Table 1 for details). Two studies reported measuring receipt and enactment^6,39^, and one study reported measuring receipt^14^ only.

#### Details of measures, sampling, and analysis

For fidelity of delivery, measures were completed by either the researcher (*n* = 18; 40.9%), provider (*n* = 11; 25%), or participant (*n* = 3; 6.8%); or both the provider and participant (*n* = 4; 9.1%), provider and researcher (*n* = 4; 9.1%), and participant and researcher (*n* = 2; 4.55%). It was not specified who completed the measures in two studies (4.55%).

For engagement, measures were completed by either the participant (*n* = 14; 30.4%), researcher (*n* = 13; 28.3%), or provider (*n* = 4; 8.7%); or both the participant and researcher (*n* = 6; 13%), provider and participant (*n* = 3; 6.5%), provider and researcher (*n* = 3; 6.5%), and the provider, participant, and researcher (*n* = 3; 6.5%).

The majority of studies (fidelity of delivery, *n* = 31; 70.45%; engagement, *n* = 42; 91.3%) did not report whether they developed their own measure or used a previously developed measure. For fidelity of delivery, eight (18.18%) used a previously developed measure and five (11.36%) developed their own measures. For engagement, three (6.5%) studies used previously developed measures and one (2.2%) developed own measures and used measures that were previously developed.

Many studies did not specify the type of scales used to quantify fidelity of delivery (*n* = 23; 52.3%) or engagement (*n* = 29; 63%). For fidelity of delivery, 12 studies (27.3%) reported using rating scales (which ranged from 3‐point scales to 10‐point scales), eight (18.2%) reported using dichotomous scales and one (2.3%) used rating scales and dichotomous scales. For engagement, 12 studies (26.1%) reported using rating scales (which ranged from 3‐point scales to 10‐point scales), three (6.5%) reported using dichotomous scales, and two (4.4%) reported using a combination of rating scales and dichotomous scales.

For both fidelity of delivery (*n* = 23; 52.3%) and engagement (*n* = 45; 97.8%), many studies did not specify how many participants they sampled. Five (11.4%) measured fidelity of delivery of all participants and 16 (36.4%) measured fidelity of delivery in a subsample of participants. Of those studies that measured fidelity of delivery in a subsample, four reported the number of sessions that they sampled, four reported the number of clinicians/sites data were sampled from, six reported the percentage of sessions that they sampled, and two did not specify how many but reported sampling some but not all participants. One (2.2%) study reported measuring engagement in a subsample of participants.

The sampling strategy used to measure fidelity of delivery included random sampling (*n* = 8; 18.2%), purposive sampling (*n* = 3; 6.8%), opportunity sampling (*n* = 1; 2.3%), stratified sampling (*n* = 1; 2.3%), self‐selected sampling (*n* = 1; 2.3%), not specified (*n* = 25; 56.8%), and not applicable for the studies that measured all participants (*n* = 5; 11.4%). No studies specified a sampling strategy for measuring engagement.

The majority of studies did not specify whether they measured fidelity of delivery (*n* = 38; 86.4%) or engagement (*n* = 35; 76.1%) in all conditions; therefore, it is likely they measured the intervention group only. Four (9.1%) reported measuring fidelity of delivery in all intervention groups, and two (4.5%) reported measuring fidelity of delivery in the intervention group only. Nine (19.6%) reported measuring engagement in all intervention groups, and two (4.3%) reported measuring engagement in the intervention group only.

For fidelity of delivery, 29 studies (65.9%) reported descriptive statistics, 11 (25%) reported descriptive and inferential statistics, and four (9.1%) did not report how they analysed the data. For engagement, 37 studies (80.4%) reported descriptive statistics and nine (19.6%) reported descriptive and inferential statistics.

Across all 66 studies, 13 (19.7%) reported using a fidelity framework.

### Reporting of psychometric and implementation qualities

#### Studies

Of all included studies, 51 (77%) reported at least one psychometric or implementation quality of their measures (38 fidelity of delivery; 86.4%, 23 engagement; 50%).

Forty‐nine studies (74.2%) reported at least one psychometric quality, and 17 studies (25.8%) reported at least one implementation quality (see Table 2 for details).

**Table 2:** Number of studies reporting psychometric and implementation qualities, across all studies (*N* = 66) and by studies reporting fidelity of delivery (*N* = 44) and engagement (*N* = 46)

	Psychometric qualities			Implementation qualities			
	Reported at least one quality	Validity	Reliability	Reported at least one quality	Practicality	Acceptability	Cost
All studies; *N* (%)	49 (74.2)	41 (62)	34 (52)	17 (25.8)	14 (21)	6 (9)	2 (3)
Fidelity of delivery; *N* (%)	37 (84.1)	31 (70.5)	29 (65.9)	12 (27.3)	11 (25)	5 (11.4)	0 (0)
Engagement; *N* (%)	21 (45.7)	16 (34.8)	10 (21.7)	9 (19.6)	6 (13.4)	2 (4.3)	2 (4.3)

#### Psychometric and implementation qualities

In total, 261 (100%) reported qualities were identified (see Table 3 for details). Of these, 215 (82.4%) psychometric qualities were reported, 41 (15.7%) implementation qualities, and five (1.9%) both psychometric and implementation qualities; 213 qualities were reported in relation to fidelity of delivery measures and 58 qualities for engagement measures.

**Table 3:** Number of times qualities were reported in total, and for fidelity of delivery and engagement

Quality	Total number of times (%)	Category	Total number of times	Fidelity of delivery	Engagement
Psychometric quality	215 (82.4)	Validity	129	100	33
		Reliability	85	75	14
		Reliability and validity	1	1	0
Implementation quality	41 (15.7)	Practicality	30	25	6
		Acceptability	8	7	1
		Cost	2	0	2
		Acceptability and practicality	1	1	0
Psychometric and Implementation quality	5 (1.9)	Reliability and practicality	1	1	0
		Validity and practicality	3	2	1
		Validity and acceptability	1	1	1
Total	261 (100)				

Note

The fidelity of delivery and engagement columns do not add up to 261 because 10 qualities were reported for both fidelity of delivery and engagement.

The most frequently reported psychometric qualities concerned the use of multiple researchers (*n* = 21: 3 data collection, 2 data analysis, 1 data entry, 3 develop measures, 11 coding, 1 validate coding frame), the validity of measures (*n* = 17: 9 valid, 8 not valid), the use of independent researchers (*n* = 16: 14 used independent researchers, 2 did not use independent researchers), reliability of measures (*n* = 11: 5 reliable, 6 not reliable), the random selection of data (*n* = 11: 9 randomly selected data, 2 did not randomly select data), and inter‐rater agreement (*n* = 9: 3 high inter‐rater agreement, 2 did not report inter‐rater agreement, 2 poor to fair, 1 fair to excellent, 1 no coder drift). Please see Table 4 for a detailed list of all psychometric qualities.

**Table 4:** Qualities, category, and number of studies qualities were reported in

Group of quality	Quality	Category	Number of studies reported in	Fidelity studies	Engagement studies
Psychometric qualities					
Use of multiple researchers	Coding	R	11	^20,26,27,29,33,34,45,51,58,64^	^47^
	Data collection		3	^6,29,31^	
	Develop measures		3	^14,26,60^	
	Data analysis		2	^10,42^	
	Data entry		1	^26^	
	Validate coding frame		1	^26^	
Validity of measures	Validated	V	9	^21,22,34,48,51^	^4,17,25,51^
	Not validated		8	^2,10,34,35,41,42,50^	^13^
Use of independent researchers	Used – coding	R	12	^20,22,26,27,29,34,38,45,51,55,63,64^	
	Not used – coding		1	^58^	
	Used – develop measures		1	^14^	
	Used – analysis		1	^42^	
	Not used	V	1	^20^	
Measurement of conditions	All conditions (result output)	V	8	^7,50^	^4,13,17,18,51,53^
	All conditions (reported)		5	^2,48,51^	^2,3,35^
	Intervention only		3	^2,24^	^24,25^
Reliability of measures	Reliable	R	6	^21,22,48^	^4,17,51^
	Not reliable		5	^2,14,23,34,50^	^2,23^
Random selection of data	Randomly selected	V	9	^31,40,51,55,57,58,63,64^	^52 (data entry)^
	Not randomly selected		2	^45,48^	
Reporting of inter‐rater agreement	Reported – high	R	3	^26,59^	^17^
	Not reported		2	^29,33^	
	Reported – poor to fair		2	^27,58^	
	Reported – fair to excellent		1	^58^	
	Reported – no coder drift		1	^26^	
Coding of sessions	A percentage	V	7	^33,45,51,55,57,58,63^	
	All		1	^27^	
Calculated inter‐rater agreement		R	8	^20,26,27,29,33,58,59^	^17^
Use of experts	Coding	V	5	^10,21,22,36,38^	
	Develop measures		1	^27^	
	Not used – coding		1	^27^	
	Checked % of data input	R	1	^10^	
Blinding	Coders	V	3	^7,26,48^	
	Not blinded		2	^2^	^52^
	Researchers		1		^15^
	Participants		1	^2^	
Measurement of content of intervention	Some aspects of intervention	V	3	^20,38^	^36,38^
	All aspects of intervention		2	^33,63^	
Problems with scoring criteria	Scoring criteria not sensitive	V	2	^20,26^	
	No success cut‐off point		1	^14^	
	Dichotomized responses reduce variability		1		^25^
	Measures may capture different aspects of fidelity		1	^26^	
Standardization of procedure	Script	V	2	^34,66^	
	Data entry		1		^52^
	Coding guidelines		1	^64^	
	Not used standardized procedure		1	^33^	
	Not used standardized measure		1		^52^
Self‐report bias		V	4	^10,26,26,30^	
		R	2	^5^	^4^
Sampling	Across all providers	V	2	^27,45^	
	Across all sites		1	^10^	
	Across all sites (purposively)		1	^33^	
	Across all participants		1	^27^	
	Balanced facilitator and gender (purposively)		1	^26^	
Audit	Data collection	R	1	^6^	
	Data analysis		1		^6^
	Coding		1	^20^	^20^
	Data entry	V	1	^23^	
	Recordings		1	^40^	
Missing responses	Missing responses	V	1		^15^
Trained researchers	Trained coders	V	3	^7,27,58^	
	Trained researcher (data collection)		1		^52^
Observation effects		V	4	^22,26,27,34^	
Use of one researcher	Coding	R	1	^38^	
	Trained observers		1	^34^	
Revised coding guidelines		R	3	^20,26,48^	
		V	1	^33^	
Team meetings		R	4	^1,6,23,36^	^23^
Recording of sessions	All sessions	V	2	^40,55^	
	% of sessions		1	^35^	
Triangulation	Method	V	2	^34,42^	
	Researcher		1	^42^	
Problems with analysis plan	Did not control for provider	V	1	^36^	
	Missing responses excluded		1	^10^	
Social desirability		V	3	^22^	^13,52^
Objective verification		V	2		^15,43^
		R	1		^12^
Used coding guidelines		R	2	^20,27^	
Analysis consideration – coded missing responses as no adherence		V	1		^15^
Independently validated coding frame		V	1	^26^	
Measurement differences – observation and self‐report		V	1	^26^	
Measurement period – year after intervention		V	1		^25^
Piloted coding guidelines		V	1	^26^	
Practice period before recording		V	1	^27^	
Pre‐specified dates for recordings		V	1	^27^	
Statistician involved in sampling (stratified)		V	1	^10^	
Training before recording may overestimate adherence		V	1	^58^	
Piloted measure		V	1	^34^	
Provided a reason for inter‐rater agreement		R	1	^27^	
Supervision		R	1	^58^	
Measures were internally consistent indicating content validity		R+V	1	^27^	
Implementation qualities					
Resource challenges	Time restrictions	P	4	^5,20,27,62^	
	Technical difficulties	P	3	^5,5,58^	
	Financial restrictions	P	2	^5,27^	
	Sharing Dictaphones	P	1	^45^	
Providers’ attitudes	Dislike paperwork	A	1	^10^	
	Fear of discouraging participants	A	1	^27^	
	Nerves	A	1	^27^	
	Report participants behaving differently	A	1	^27^	
	Positive attitudes	A	1	^42^	
	Additional work	A	1	^62^	
	Not enthusiastic	A	1	^62^	
Measurement of content of intervention	Telephone calls not assessed due to difficulty	P	1	^38^	
	Measure cannot capture non‐verbal data	P	1	^20^	
Problems with documentation	No record of responses	P	2	^10,58^	
	Providers did not document everything		1	^10^	
	No record of refusals	A+P	1	^27^	
Missing responses	Missing responses	P	1	^10,10 (different aspects)^	
Problems with sampling	Low recruitment	P	1	^60^	
Problems with analysis plan	Analysis not feasible	P	1	^10^	
Incentives	Incentives used	P	2		^15,52^
	Incentives required	P	1	^62^	
Feedback to providers		P	2	^21,27^	
Feedback delay		P	1	^38^	
Forgetting to return data		P	1		^15^
Logbook showed that not all steps were applied		P	1	^42^	
Paper and digital version of measures given		P	1		^5^
Need simpler coding guidelines to achieve agreement		P	1	^27^	
Reviewed fidelity after trial		P	1	^45^	
Participants – dislike paperwork		A	1		^15^
Did not do a cost analysis		C	1		^13^
Cost of materials		C	1		^37^
Both psychometric and implementation qualities					
Problems with scoring criteria	Lack of clarity on items	V+P	1		^25^
Missing responses	Missing responses	V+P	1	^58^	
Use of one researcher	Data collection	R+P	2	^5^	^52^
Problems with sampling	Selection bias	V+A	1	^2^	^2^
	Not randomly selected	V+P	1	^27^	

Notes

This table is ordered by the number of studies that reported a quality that fits into the ‘group of quality’ column (e.g., ‘use of multiple researchers’). Most frequent → Least frequent. The numbers in this table will not add up to the total number of studies included, as some studies included information on multiple qualities.

R = reliability; V = validity; A = acceptability; P = practicality; C = cost.

The most frequently reported implementation qualities concerned resource challenges (*n* = 10: 1 sharing Dictaphones, 4 time restrictions, 2 financial restrictions, and 3 technical difficulties) and providers’ attitudes (*n* = 7: 1 dislike paperwork, 1 fear of discouraging participants, 1 nerves, 1 report participants behaving differently, 1 positive attitudes, 1 additional work) (see Table 4 for a list of all qualities).

## Discussion

### Key findings

Fewer than half of the reviewed studies measured both fidelity of delivery of and engagement with complex, face‐to‐face health behaviour interventions. Measures covered observation, self‐report, and intervention records. Whilst 73% reported at least one psychometric quality, only 26% reported at least one implementation quality.

### How findings relate to previous research

The measures used to measure fidelity of delivery of, and engagement with, complex, face‐to‐face health behaviour change interventions were consistent with previous recommendations of using observational or self‐report measures to monitor fidelity of delivery, and self‐report measures to monitor engagement (Bellg *et al*., 2004; Borrelli, 2011; Burgio *et al*., 2001; Carroll *et al*., 2007; Schinckus *et al*., 2014). A similar percentage of studies used observational and self‐report measures to measure fidelity of delivery, despite observational measures being recommended as the gold‐standard measure and the reported limitations of self‐report measures (Bellg *et al*., 2004; Borrelli, 2011; Breitenstein *et al*., 2010; Lorencatto *et al*., 2014; Schinckus *et al*., 2014). Intervention records (e.g., attendance or homework) were also used to measure engagement. Intervention records can be considered an objective measure of receipt (Gearing *et al*., 2011; Rixon *et al*., 2016) and participation (Saunders, Evans, & Joshi, 2005). However, these measures are limited by their inability to monitor how much participants understand and use the intervention. Other recommended and potentially more objective measures, for example, asking participants to demonstrate skills (Burgio *et al*., 2001), were not adopted by any study in this review. Perhaps these findings demonstrate that measures need to be easy to use and acceptable to respondents and researchers in order to be selected for use. This explanation is consistent with previous studies which suggest that observational measures are perceived to be more expensive, time‐consuming and difficult to use (Breitenstein *et al*., 2010; Schinckus *et al*., 2014). Many studies used measures of fidelity of delivery and engagement specific to one intervention, and therefore, generalizability is limited (Breitenstein *et al*., 2010).

This review found that three quarters of studies reported at least one quality of their measures. This finding demonstrates that the reporting of psychometric qualities in the complex, face‐to‐face health behaviour change interventions included in this review, may not be as infrequent as previously suggested in different populations (Baer *et al*., 2007; Breitenstein *et al*., 2010; Maynard *et al*., 2013; Rixon *et al*., 2016). However, not all studies reported psychometric qualities, and fewer reported implementation qualities, despite the importance of psychometric and implementation qualities (Gearing *et al*., 2011; Glasgow *et al*., 2005; Holmbeck & Devine, 2009; Lohr, 2002; Stufflebeam, 2000). The reporting of psychometric and implementation qualities provides information which allows the reader to determine whether the findings are trustworthy and representative. Given this, it is difficult to draw conclusions with high certainty about how well interventions have been delivered or engaged with. This, in turn, makes it difficult to draw conclusions about intervention effectiveness.

The psychometric qualities that were most frequently reported were those recommended by previous research; examples of these are the use of multiple, independent researchers to reliably rate a random percentage of sessions for fidelity of delivery (Bellg *et al*., 2004; Borrelli, 2011; Lorencatto *et al*., 2014). However, some qualities which are recommended by research were not frequently reported; an example of this is routine audio‐recording (Gresham, Gansle, & Noell, 1993; Miller & Rollnick, 2014). The implementation qualities that were most frequently reported were those concerning resources (including time constraints, financial constraints, and technical difficulties) and providers’ attitudes towards measures. These findings could explain why missing responses were reported in some of the studies included in this review (Arends *et al*., 2014; Chesworth *et al*., 2015; Dubbert, Cooper, Kirchner, Meydrech, & Bilbrew, 2002; Thyrian *et al*., 2010) and health care research (Shrive *et al*., 2006). Providers may not return audio‐recordings (Weissman, Rounsaville, & Chevron, 1982) or checklists, if they feel uncomfortable with audio‐recording or if they are overwhelmed with paperwork.

### Limitations

The aim of this review was to identify a range of studies that met the criteria and reported fidelity of delivery and/or engagement in enough depth to be able to draw conclusions about the reporting of fidelity of delivery and/or engagement measures. To identify as many studies as possible, a comprehensive search was conducted, which included contacting experts and authors to identify further relevant articles that may have been missed by the search strategy. However, we will not have identified articles that did not report monitoring fidelity of delivery or engagement in titles, abstracts, or keywords. A further reason why relevant articles may have been missed is that many terms are used interchangeably in fidelity research and we may not have captured all of these terms in the search strategy. We only included articles that reported a clear fidelity of delivery or engagement measure or outcome. As is the case with many systematic reviews, the search is inevitably limited to its date cut‐off. However, future use of natural language processing, ontologies, and machine learning (Larsen *et al*., 2016) will enable more ongoing updating when aggregating review evidence (see www.humanbehaviourchange.org).

The findings from this review consider the reporting of qualities and not the actual quality of measures. The review findings do not consider strengths or weaknesses of these qualities nor how much weighting should be given to each quality when designing fidelity of delivery and engagement measures. This is an area that could be investigated, building on the current review.

### Implications

There are three main implications of these review findings for researchers and intervention developers:

1. The need to fully report details of fidelity of delivery and engagement measures. The findings from this review demonstrated that many studies did not specify details about the sampling or analysis method used in developing measures of fidelity of delivery and or engagement. If this information is not available, evaluation and replication are difficult to achieve.
2. The need to report both psychometric and implementation qualities for fidelity of delivery and engagement measures. The reporting of psychometric and implementation qualities would be helpful to researchers who are aiming to measure fidelity of delivery or engagement. This information would allow evaluations of what measures and procedures may be feasible.
3. The need to develop high‐quality measures of fidelity of delivery and engagement that are acceptable and practical to use but also reliable and valid. Both psychometric and implementation qualities of measures are relevant when selecting, developing, and reporting measures.

If implemented, these steps could help to strengthen the quality of fidelity of delivery and engagement data and the interpretation of intervention effectiveness.

### Future research

Further research is needed to evaluate the importance and weighting of each quality when designing fidelity of delivery and engagement measures. One way to do this could be to conduct a Delphi study with experts in intervention fidelity and engagement. This systematic method could be used for building a consensus (Hsu & Sandford, 2007) regarding which psychometric and implementation qualities are most important, and which qualities should be given the most weighting when developing and evaluating fidelity of delivery and engagement measures. This information could then to be used to inform the development of measures of fidelity of delivery and engagement that are reliable, valid, acceptable, and practical. Future systematic reviews could explore the qualities of fidelity and engagement measures reported in qualitative studies.

### Conclusion

Fewer than half of the reviewed studies measured both fidelity of delivery of and engagement with complex, face‐to‐face health behaviour change interventions. Measures covered observation, self‐report, and intervention records. Whilst 74% reported at least one psychometric quality, only 26% reported at least one implementation quality. Findings suggest that implementation qualities are reported less frequently than psychometric qualities. The findings from this review highlight the need for researchers to report measures of fidelity of delivery and engagement in detail, to report psychometric and implementation qualities, and to develop, use, and report high‐quality measures. This would strengthen the quality of fidelity of delivery and engagement data and the interpretation of intervention effectiveness.

## Funding

Holly Walton's PhD is funded by the Economic and Social Research Council (ESRC) Doctoral Training Centre (Grant reference: ES/J500185/1). Ildiko Tombor's post is funded by a programme grant from Cancer Research UK. The funding bodies played no role in designing, conducting, analysing, interpreting or reporting the results of the review.

## Conflict of interest

The authors declare no conflict of interests.

## Compliance with ethical standards

This research is a review and did not involve research with human participants or animals.

## Supporting information


**Appendix S1.** Search strategy.
**Appendix S2.** Characteristics of included studies.
**Appendix S3.** The proportion of studies which measured fidelity of delivery, engagement, or both.
**Appendix S4.** Details extracted from the papers on fidelity of delivery, and engagement methods and results.
