# Supplementary material for: Measures of fidelity of delivery of, and engagement with, complex, face‐to‐face health behaviour change interventions: A systematic review of measure quality
Source: Br J Health Psychol. 2017 Aug 1;22(4):872–903. doi: 10.1111/bjhp.12260 (PMC5655766; doi:10.1111/bjhp.12260)
Supplement: Supplementary file 1 — Appendix S1. Search strategy. Appendix S2. Characteristics of included studies. Appendix S3. The proportion of studies which measured fidelity of delivery, engagement, or both. Appendix S4. Details extracted from the papers on fidelity of delivery, and engagement methods and results. [file BJHP-22-872-s001.docx]

Appendix 1: Search strategy

The initial search strategy used to identify records

1. “Fidelity of delivery” OR “Treatment fidelity” OR “Treatment delivery” OR “Delivery” (In title and abstract)
2. “Engagement” OR “Treatment receipt” OR “Treatment enactment” (In title and abstract)
3. “Program evaluation” OR “Process evaluation” (abstract)
4. Complex intervention OR Multicomponent intervention OR Multiple intervention components
5. “Behavior change” intervention OR “Behaviour change” intervention
6. Health or “healthcare” (In title and abstract)
7. Combine 1 and 2 with OR
8. Combine 7 and 3 with OR
9. Combine 4 and 5 and 6 with AND
10. Combine 8 and 9 with AND

| Appendix 2: *Characteristics of included studies* | | | | | | |
| --- | --- | --- | --- | --- | --- | --- |
| **Authors (and date)** | **Methods** | **Participants** | **Facilitator characteristics and training** | **Description of intervention** | | |
|  |  |  |  | **Intervention groups** | **Target behaviour** | **Delivery mode, sessions, duration** |
| **Apter et al (2011) ^1^** | Randomized controlled trial | Primary care and asthma practices. 333 (165 in PS, 168 in control) adults with asthma | Research coordinators (college graduates). 3-week training using manuals | Problem solving intervention. Control: Standard asthma education. | Improve adherence to asthma treatment | 4 face-to-face 30 minute sessions. Control: Four 30 min sessions. |
| **Arends et al (2014) ^2^** | Process evaluation of cluster randomized controlled trial | Occupational health services in Netherlands. 109 workers in intervention, 64 process evaluation - | 126 physicians. 2-day training. Experienced trainers. Three feedback moments. | SHARP intervention. Control group: care as usual. | Minimise recurrent sickness absence | Two to five face-to-face consultations within 3 months. |
| **Baker et al (2001) ^3^** | Randomized controlled trial | Community based. 56 participants (over 55) (23 control/23 intervention) with knee osteoarthritis. | Not provided | Home based progressive strength training program and attention control group. | Exercise | Face-to-face. 3 times per week for exercise. 2 home visits for 3 weeks and once every fortnight after 12 weeks. |
| **Bailey et al (1990) ^4^** | Repeated measures design | Pulmonary Medicine Clinic, individuals with asthma diagnosis (101 usual care, 124 intervention) | Health educator delivering programme. | Self-management intervention vs usual care | Asthma self-management practices | One to one face-to face counselling session (1 hour) |
| **Binkley et al (2014) ^5^** | Pilot study (pre/post) | 12 homes (2 cohorts). 25 residents with IDD. 21 caregivers | Trained at baseline. No information. | Oral health strategy with four components. | Oral hygiene practices | 2 face-to-face coaching sessions (1.5-2 hours, 30 min – 2 weeks apart) |
| **Black (2014) ^6^** | Quasi experiment (pre/post) | Community setting. 120 caregivers of older adults. | Bachelor level social worker. 8 hr training | CAREs intervention. 4 phases. | Caregiver skills | Face-to-face, 3-12m |
| **Brug et al (2007) ^7^** | Randomized controlled trial | 9 home care organisations in Netherlands. 209 patients with diabetes. | 37 dieticians (bachelor’s degrees, (2 to 20 years’ experience), 2 days training by senior dieticians in MI | MI intervention and no MI intervention. | Changes in dietitians counselling style | 2 face-to-face one week sessions. Participants = 4/5 counselling sessions (30-35 mins /15mins) |
| **Butler et al (2012) ^8^** | Randomised controlled trial | 66 GPs in Wales, 142 clinicians. (34 practices in intervention) | Clinicians (intervention: 127 clinicians, control 135 clinicians) | Blended learning experience and usual care. | Reduce antibiotic dispensing | Face-to-face and online |
| **Campbell et al (1998) ^9^** | Randomized controlled trial | 19 General practices in N.E Scotland. 673 patients in intervention, 670 in control | 28 District nurses/practice nurses. Day and a half training on manual and techniques. Phone support. | Clinic visits and control. | Secondary prevention (aspirin use/BP management, exercise, diet, smoking) | Face-to-face clinics for a year (first session 45 mins, follow up 20 mins) |
| **Chesworth et al (2015) ^10^** | Open cluster randomized feasibility trial | 12 NHS stroke services (England/Wales). 413 stroke patients | Intervention protocol. Online and face-to-face training | Intervention, Supported implementation, Usual care. | Management of UI after stroke | Face-to-face |
| **Cheung et al (2015) ^11^** | Quasi experiment (pre/post) | 11 NGOs. 243 cases. Caregivers in Hong Kong (6m+), and individuals with dementia | 85 providers (social workers 1yr+ experience). Training: supervised, video recordings, manual | REACH II. Individualized psychoeducational and skills training based NPI. | Caregiver skills and reduce behavioural problems | Nine in home face-to-face 1 hr sessions and three 0.5 hr telephone sessions |
| **Dannhauser et al (2014) ^12^** | Open label study (pre/post) | Home/community based. 70 individuals with MCI | Training and materials for tutors | ThinkingFit programme. (Exercise, group based CST, individual CST) | Exercise | 12 face-to-face sessions (one per week) 20-45 mins. + 2.5hr sessions + 30m x 3 per week |
| **DeWalt et al (2006) ^13^** | Randomised controlled trial | University medicine practice. 127 participants using furosemide (62 intervention/62 control) | Clinical pharmacist/health educator | Education session vs control group (general education pamphlet) | Self-management skills | Hour face-to-face session. 5-15 min follow up calls |
| **Driessen et al (2010) ^14^** | Process evaluation of a cluster RCT | 4 Dutch companies (railway, airline, steel, university). Depts. With 3000 workers, 2 years 20hours+ | Ergocoaches. 40 implementers followed 4-hour training | Stay@work intervention. Control | Reduced episodes of neck and back pain | One 6hr face-to-face working group meeting. Three short movies. |
| **Dubbert et al (2002) ^15^** | Randomized controlled trial | Medical Centre. 181 participants (59 PC, 62 PC and AC, 60 NC) | None mentioned | Clinic based activity counselling and follow up calls (x20, x 10 or none). | Exercise | One Face-to-face session then telephone (x20, x 10 or none) |
| **Duff et al (2013) ^16^** | Uncontrolled intervention + process evaluation (pre/post) | 250 bed private hospital in Sydney, Australia. 85 nursing staff. 98 patients | Registered nurse – expert VTE knowledge (20+yrs). 2-day workshop | Educational outreach visits. | Provide prophylaxis based on VTE risk | 1 face-to-face educational visit |
| **Duncan et al (2003) ^17^** | Randomised controlled trial | One HF clinic, participants with an ejection fraction of 40% or less, receiving pharmacologic therapy | None mentioned. | Exercise with adherence facilitation, exercise only. | Exercise | Face-to-face 3 times a week then asked to do two /three times a week at home. |
| **Ettinger et al (1997) ^18^** | Randomised single blind clinical trial | 2 clinical centres at universities. 439 Community dwelling adults over 60 with knee osteoarthritis | Not provided. | Aerobic exercise training, resistance exercise training, health education control | Exercise | 8 face-to-face 1 hour sessions a week (10-15 people). Home based (4 visits and 6 calls). |
| **Farmer et al (2007) ^19^** | Three arm open parallel group randomized trial | General practices. 453 patients with type 2 diabetes (152 usual care/150 less intensive/ 151 more intensive) | Research nurses. Training: Psychological theory and trained in behaviour change techniques. | Less intensive self-monitoring, more intensive self-monitoring, usual care. | Regular blood glucose monitoring | Face-to-face (after randomisation, one, three, six and nine months. |
| **French et al (2015) ^20^** | Cluster randomized trial | 36 GP’s. 7 practices (53 GPs control, 45 practices 59 GPS to intervention). Participants with acute non-specific LBP | Each session = 2 or 3 facilitators. 4 GPs, 2 were allied health clinical professionals | Two session workshop. And control group. | Reducing referrals of patients with back pain for x-ray | Two session workshops (3 hour) face-to-face sessions. |
| **Gabbay et al (2013) ^21^** | Randomized controlled pragmatic trial | 12 primary care clinics. Patients with type 2 diabetes (313 in control, 232 in intervention) | Three nurses. Standardised training by HCPs certified in M. (80 hour) | Intervention vs usual care. | Self-management behaviours | Hour long face-to-face sessions. Phone call/email. Control visits every 3m |
| **Goyder et al (2014) ^22^** | Process evaluation of three arm parallel group randomised controlled trial. | Deprived areas of Sheffield between 40-64 years, not achieving recommended exercise. 282 randomised (control 96, mini booster, 92, full booster 94). | Research assistants trained by MI trainer. 6 days formal training, supervision. 3 years’ experience + physical activity experience | MI intervention, control, no intervention. | Physical activity | Full booster = 2 face-to-face sessions (20-30 mins), mini booster two physical activity MI consultations by telephone |
| **Griffin et al (2010) ^23^** | Process evaluation | 12 sites across US. 4689 participants in 328 groups. | Multiple trainings for program staff (phone calls and meetings). | Active for Life | Physical activity | 20 face-to-face weekly classes. One to one counselling and eight telephone calls. |
| **Grubbs et al (2015) ^24^** | Secondary analysis of an RCT. | 17 primary care clinics in California, Washington and Aransas (4 sites). 1004 patients –PD, GAD, SAD, PTSD. 501 usual care 503 intervention | Care managers: master’s degrees in social work/nursing. Supervised by clinical psychologist | Collaborative care intervention. Medication CBT both or neither. Usual care | Reduce anxiety symptoms | 8 face-to-face CBT sessions, optional monthly relapse prevention by phone |
| **Hankonen et al (2014) ^25^** | Randomised controlled trial | 34 general practise in 4 areas of England. Participants with type two diabetes. 229 intervention, 239 control | 3 trained lifestyle facilitators. Two background in nursing, social work. Training = 7 days | Intensive diabetes treatment plus behaviour change intervention (Addition plus). Control: Diabetes treatment only. | Physical activity | 1 year face-to-face and telephone. 1 hour introduction meeting, 6 30 min meetings and four brief phone calls. |
| **Hardeman et al (2008) ^26^** | Fidelity analysis of ProActive Trial | UK, 52 participants from Proactive trial. 27 participants. | Dietician, two nurses and physical fitness instructor. 5-day training + manual | Three trial arms: Brief advice, face-to-face intervention, distance intervention. | Physical activity | Face-to-face intervention. 4 sessions at participants’ home. Phone calls and monthly contact |
| **Harting et al (2004) ^27^** | Quality assessment of a randomized controlled trial | University hospital Maastricht and 35 GPs of 25 practice units. 2743 high risk of cardiovascular event within 10 year patients. | 10 cardiologists and 35 GP’s. Four health advisor. 2-day practical course + 2-day training. Feedback sessions | High risk intervention. | Reduce behavioural cardiovascular risk factors | No more than 6 face-to-face consultations (45 then 30min) |
| **Hermens et al (2001) ^28^** | Nationwide prospective cohort study | Random sample of 1/3 of all 4758GPs in Netherlands. 1586 GPs | Educational materials. Support by outreach visitors + training | National prevention programme. | Adherence to cervical cancer screening guidelines | Face-to-face |
| **Holtrop et al (2015) ^29^** | Mixed methods study | 5 practices in Michigan USA. Intervention practices in a larger intervention | 2 researchers (co-investigator/research assistant) | Intervention: Chronic care model. Control group. | Diabetes management | Face-to-face |
| **Hunt et al (2001) ^30^** | Process evaluation of a nutrition RCT | 6 group practices (Harvard Pilgrim Healthcare). 230 patients 274 in control | 28 providers (intervention), 50 (control). Telephone counsellors (master’s students). 2 4h training workshops + weekly meetings | EatSmart intervention. Control group. | Health behaviours (diet) | Brief face-to-face PCP endorsement, 2 counselling calls, consultation with dietician. |
| **Yu-Yahiro et al (2009) ^31^** | Randomised controlled trial. | Home based. 91 Women over 65, with hip fracture | Trainer randomized. Previous experience. | Exercise program, plus or motivational component, and usual care. | Exercise | 5 days a week face-to-face exercise sessions. 3 trainer supervised exercise sessions per week (2 months) |
| **Jansink et al (2013) ^32^** | Cluster randomised trial. | 58 general practices in Netherlands. Patients with type 2 diabetes. | Nurse led. 4 half day training session | Intervention group and usual care. | Lifestyle behaviours | Face-to-face |
| **Keith et al (2010) ^33^** | Mixed methods sequential exploratory design of quasi experiment | 4 medical centres (2 tertiary, 2 primary). 457 patients with chronic heart failure. | 18 clinicians | Nurse practitioner case management program. | Inpatient resource use | Face-to-face |
| **Lawrence et al (2014) ^34^** | Non-randomised controlled trial | Sure start centres. All play, family support and community development workers. 148 practitioners | Training delivered by researchers experienced in behaviour change. | Intervention vs no healthy skills training. | Health behaviour change – client centred skills | Three face-to-face 3-hour group sessions over 3-5 weeks. |
| **Lazovich et al (2000) ^35^** | Randomised controlled trial | 6 primary care clinics in Washington. 28 practices, Individuals with an appointment (1010 intervention, 1111 control). | Brief (20/30 min) training session. Introduce intervention | Intervention (written materials physician verbal message, follow up support) | Diet | Face-to-face |
| **Lobb et al (2004) ^36^** | Randomised controlled trial | 14 health centres in low income multi ethnic neighbourhoods 1088 participants aged 18-75 | 97 physicians, nurse practitioners and physician assistants. Previous experience. 16 hrs training, 8hr social training, ongoing support, | Healthy directions health centre’s intervention. Control group. | Health behaviours (diet, physical activity) | Face-to-face clinician endorsement (1hr). 2 health counselling sessions. 4 telephone calls. |
| **Matei et al (2015) ^37^** | Mixed methods analysis of an uncontrolled trial (pre/post) | Sheltered housing and community and faith centre. Participants (more than 6 hours daily sitting times, 60-75 years old. | None mentioned | Intervention. | Reduce sedentary behaviours | Face-to-face |
| **McCarthy et al (2015) ^38^** | Mixed method process evaluation (pre/post) | HF clinic within large urban hospital. 20 ethnic minority adults 18+ with stable HF (60% male) | 3-day workshop with feedback | Exercise counselling, 12-week telephone follow up, daily diary for self-monitoring. | Exercise | 1 face-to-face exercise counselling session. 12 weeks of calls |
| **McCurry et al (2012) ^39^** | Pilot Randomised controlled trial | 37 homes. 47 residents with dementia and sleep problems | MSW trainer 15 years’ experience. 2r training with PI, weekly supervision, tape review | Sleep education program. Usual care control. | Improving sleep | 4 face-to-face sessions. |
| **McGillion et al (2008) ^40^** | Randomized controlled trial | 3 university teaching hospitals in Canada with large cardiac outpatient programs. 130 CSA patients living in community, 66 CASMP | Facilitator manual. Registered nurse (8-15 patients) | Chronic angina self-management program (group). Wait list control. | Self-management skills | Weekly 2 hr face-to-face sessions for 6 weeks |
| **McNamara et al (2015) ^41^** | Complex educational intervention | 10 community pharmacies in Victoria/Tasmania (5 rural/5 metropolitan). 70 patients aged 50-74 years taking high BP medicine. | 12 experienced pharmacists. Instructed in health education and behaviour change | Clinical report and action plan, report highlighted risk, suggest goals. | Reduce CVD risk (health behaviours) | Five face-to-face sessions (30 mins then 15 mins) at monthly intervals |
| **Metzelthin et al (2013) ^42^** | Process evaluation of cluster Randomized controlled trial using mixed methods | 6 GP practices in South Netherlands. 194 frail older people (77.49, 55% female) | 12 GPs and 7 practice nurses. Bachelor degree, expertise, effective communication and problem solving. 3m meeting supervision and feedback. | Prevention of care approach. Usual care. | Self-management skills | Face-to-face |
| **OBarzanek et al (2007) ^43^** | Randomised trial | Four clinical centres. Healthy adults, 810 participants | Trained certified staff | 2 treatment groups: behavioural intervention and Established Plus DASH, advice control group. | Physical activity and healthy eating | Face-to-face |
| **Ockene et al (2012) ^44^** | Randomised intervention | Massachusetts. 312 Latino participants who are at high risk for diabetes (150 usual care, 162 intervention | 3 Spanish speaking community individuals delivered intervention (undergraduate in nutrition). Training = role play and mock sessions + annual booster sessions led by a behavioural psychologist/dietitian) | Lifestyle intervention or usual care | Diet/physical activity | 3 individual and 13 face-to-face group sessions over 12m. (Session 1 1.5 hours, rest 1 hour). |
| **Olsen et al (2012) ^45^** | Single centre randomized controlled trial | Single centre. Diagnosis of OSA, clinical recommendation for CPAP. 106 participants | Three nurses with 2-16 years sleep medicine training. Full day training. Regular supervision | MINT intervention: vs standard care. | Sleep | 30 min face-to-face session. |
| **Osborn et al (2010) ^46^** | Randomised controlled trial. | Primary care clinic, 129 participants | 40 hours training. Bilingual medical assistant. | Intervention vs usual care. | Health behaviours | 90 min face-to-face session. |
| **Pettman et al (2008) ^47^** | Evaluation of parallel Randomised controlled trial | Community based. South Australia. 153 overweight volunteers’ Metabolic syndrome. 103 intervention, 50 control. | Leader’s manual. PowerPoint slides. Supporting materials. | Shape up for life intervention. Control group. | Diet/physical activity | 16 face-to-face weekly lifestyle sessions (2hrs) and one exercise session per week (45-60 min) |
| **Pill et al (1998) ^48^** | Parallel process study of a randomised controlled trial | 19 GPs in South Glamorgan. Patients diagnosed with type 2 diabetes over 1 year aged 18-70. | GP research nurse and psychologist. Training = open exploration. | Intervention vs control group. | Encourage clinicians to negotiate individual care plans | Face-to-face (at least 2 training sessions, 3 hours) |
| **Roy-Byrnes et al (2010) ^49^** | Randomised controlled effectiveness trial | 17 Primary care clinics in 4 US cities. | 14 ACS personnel (11 women, 3 men (social workers/MSc psychologists, doctoral psychologist). 4 familiar with CBT 7 previous training | CALM intervention vs usual care. | Reduce anxiety symptoms | 6 to 8 weekly face-to-face sessions for CBT. Single medication training. Telephone/email consultations |
| **Saunders et al (2014) ^50^** | Evaluation of a group Randomised controlled trial | Community based project in 128 African American churches. 37 intervention, 31 control | Pastor. Full day training, cook training, monthly mailings and technical assistance | FAN Intervention. Delayed churches (15m) | Physical activity, healthy eating | Face-to-face |
| **Skidmore et al (2014) ^51^** | Non-randomised pilot study | 2 inpatient rehabilitation units within an academic health centre. 10 individuals with an acute stroke diagnosis. 5 in intervention | Trained Occupational therapists (one in each condition). Manualised procedures | Strategy training. Attention control session in addition to usual rehabilitation care. | Activities of daily living | Daily face-to-face sessions, five days per week 30-40 min) for whole rehabilitation |
| **Slade et al (2015) ^52^** | Cluster randomized controlled trial | 2 mental health NHS trusts in England. 210 participants with a clinical diagnosis of psychosis in intervention, 193 in control | 12 hours (personal recovery), 16 hours (recovery coaching) training, telephone support and booster sessions, 6 reflection groups, reflective tool | REFOCUS intervention and usual treatment. | Staff behaviour to increase focus on values, preferences, strengths and goals of patients with psychosis. | Face to face 1 year intervention. |
| **Smith et al (1997) ^53^** | Randomized pilot study | 22 Women with NIDDM aged 50 or over | Team of interventionists (nutritionist, three psychologists and an exercise psychologist. | Behavioural weight program vs standard behavioural weight control program. | Exercise, diet | 16 face-to-face sessions. Intervention = 3 MI sessions. |
| **Smith et al (2010) ^54^** | Observational study | 14 GPs in a mixed urban rural area of Scotland. 1584 participants between 18 and 64 – low mood/depression /adjustment disorder | Mental health clinicians, psychology graduates, primary care liaisons (experienced nurses/OTs). 30hr training + 2h weekly supervision | Doing well intervention (health centres). | Antidepressant use, alcohol, exercise | Face-to-face (50 min) |
| **Stanley et al (2013) ^55^** | Pilot randomised controlled trial | Outpatient clinics at medical centre. 182 Participants with a dementia diagnosis and possible anxiety | Masters level graduate students and a pre-doctoral intern. Extensive training. | Peaceful mind vs usual care. | Reduce anxiety symptoms (behavioural skills) | 12 weekly face-to-face in home sessions over initial 3 months and 8 telephone appointments |
| **Suzuki et al (2012) ^56^** | Randomised controlled trial. | Community centre in Japan. 50 Elderly individuals (over 65) with MCI. | Two physiotherapists and three well trained instructors. | Multicomponent intervention vs education control group. | Exercise | 90 min per day, 2 days a week, 80 times over 12 month face-to-face. Control: 3 education classes |
| **Thyrian et al (2007) ^57^** | Evaluation of Randomised controlled trial | Maternity wards of six hospitals in Germany. 163 Women who smoke before pregnancy (mean 26.28) | Counsellors trained experts in MI – weekly supervision. | Counselling vs usual care. | Smoking cessation | 1 face-to-face counselling session and two follow up counselling sessions by phone. |
| **Thyrian et al (2010) ^58^** | Evaluation of a randomised controlled trial | Maternity ward of six hospitals in Germany. 84 women smoked before pregnant. | Four counsellors- trained experts in MI. Weekly supervision meetings. | Counselling vs usual care. | Smoking cessation | 1 face-to-face counselling session (45 min) and telephone support. |
| **Tomasone et al (2014) ^59^** | Process evaluation within CMCL intervention | Within CMCL in Canada. 97 HCPs | Delivered by HCP and physically active individual with a disability. 66 were HCP 26 were not | Changing minds, changing lives. | Increase professional behaviour (prescribing physical activity) | Single face-to-face sessions (1hr) |
| **Van de Glind et al (2012) ^60^** | Mixed methods multiple case study – multicentre Randomised controlled trial | 5 purposively selected healthcare settings in Netherlands. Main trial = 11 sites. 53 leg ulcer patients in program, 45 evaluation period | 12 women nurses. Nurse scientist, clinical psychologist, MI trainer delivered training | Lively legs program. Usual care. | Health behaviour (physical activity/adherence) | 6 face-to-face sessions (45-60 min then 20 mins) |
| **Wallace et al (1998) ^61^** | Randomised controlled clinical trial. | Senior community centre. 100 adults Over 65 and ambulatory | None mentioned. | Multiple risk factor intervention. | Exercise, nutrition, safety, smoking, alcohol | 30-60 min visit, 60 min exercise session, 3 weekly sessions (6m) |
| **Weinberger et al (2002) ^62^** | Randomised controlled trials | 36 community drug stores. Participants with COP/asthma and specific prescription | 4-hour pharmacist training | Intervention vs peak flow monitoring control group | Pharmacist behaviours | Face-to-face |
| **Welch et al (2011) ^63^** | Randomized trial | Large hospital medical centre. 234 Participants 30-70 years, poor control blood glucose. | Two days of workshop training and group conference call session, Individual phone feedback. | Standard education, computer alone, MI alone, MI with computer. | Blood glucose control | One hour session followed by three 30 min sessions. |
| **West et al (2007) ^64^** | Randomized controlled clinical trial | Birmingham. Women with type two diabetes. | Intervention: clinical psychologists. Control: Master’s degree health educators | Group based behavioural obesity treatment, plus experimental condition: MI/behavioural obesity/attention control. | Glycaemic control | All: 42 face-to-face session weight management, weekly for 6 months, biweekly 6 months and monthly (45 mins). Control: Matched to intervention |
| **Wieland et al (2012) ^65^** | Pilot study | Rochester, Minnesota, Community based participatory research approach. 45 women (22-68) in communities | Trained community focus group mediators | Fitness program – 2 classes per week, exercise, nutrition components. | Physical activity, diet | Two face-to-face classes per week (90 min) |
| **Windsor et al (2014) ^66^** | Process evaluation of a non-randomised matched comparison group design | All RFTS clients reported smoking at screening and were over 18. 1303 clients (age 34.3 vs 23.8). | SCRIPT training (performance reports and quality improvement process). | SCRIPT program vs control group | Smoking cessation | Face-to-face sessions. |

Appendix 3: *The proportion of studies which measured fidelity of delivery, engagement, or both.*

| Measured | Number of studies (%) | Study reference numbers |
| --- | --- | --- |
| Fidelity of delivery and engagement | 24 (36.4) | ^2,5,6,10,11,14,16,19,21,23,24,29,30,35,36, 38,39,40,41,42,48, 51, 55, 64^ |
| Fidelity of delivery | 20 (30.3) | ^1,7,20,22, 26,27,28,31,33,34,45,49,50, 57,58,59,60,62,63,66^ |
| Engagement | 22 (33.3) | ^3,4,8,9,12,13,15,17,18,25,32,37,43,44,46,47,52,53,54,56,61,65^ |
| Total | 66 (100) |  |

Appendix 4: *Details extracted from the papers on fidelity of delivery, and engagement methods and results*

| **Study** | **Fidelity framework used (if applicable)/ terms and definitions** | **Fidelity / engagement** | **Method** | **Results** |
| --- | --- | --- | --- | --- |
| **Apter et al (2011) ^1^** | None mentioned | Fidelity | **Procedure & measures:**   1. Observation of research-patient interactions by project managers (early stage) 2. Unannounced observation (later stage)   Measures not described  **Sample** – Not provided  **Analysis method:** Procedures and problems discussed at weekly team meetings with primary investigator. Percentages used to quantify | 100% fidelity to the protocol |
|  |  | Engagement | Not measured | Not measured |
| **Arends et al (2014) ^2^** | Based on Steckler and Linnan’s (2002) framework. The authors define ‘fidelity’ (quality), ‘dose delivered’ (completeness), ‘dose received’ (exposure), as is defined in Steckler and Linnan’s (2002) model | Fidelity | **Procedure & measures:** Self-report questionnaires (physician and participant) collected at 3m follow up to assess dose delivered in intervention and control group (questions about number and type of assignments and whether stimulated them) and fidelity in intervention only (core components: number of participants who received two key elements). Dose delivered scored 5 point Likert scale (totally disagree-totally agree) and fidelity scored yes/no.  **Analysis method:** Descriptive statistics, regression analyses, multilevel analysis. Used participants’ blinded responses | Intervention vs control. Assignments received from OP (73% vs 8%): 1 (66%), 2 (54%), 3 (48%), 4 (48%), 5 (36%). OP being involved 3.9 vs 3.5, OP stimulating making own decisions 3.8 vs 3.6. Topics related to RTW: Problems at work 84% vs 63%, possible opportunities 49% vs 27%, solutions 58% vs 34%, realising opportunities 45% vs 36%, who can help 55% vs 22%, action plan 25% vs 25%, evaluation of RTW 46%vs 55%. Physician report: 63% delivered 2 consultations with OP and delivered first assignment. Participant report: 64% delivered 2 consultations with OP and first assignment completed. >2 consultations with OP and first assignment completed by participant 64% |
|  |  | Engagement | **Procedure & measures:** Self-report questionnaire (physician and participant) collected at 3m follow up in intervention and control group to assess dose received (number and type of assignments completed by participants, number and type of topics discussed, number of participants who had two consultations with physician and completed first assignment).  **Analysis method:** Descriptive statistics, multilinear regression, multilevel analysis | Assignments completed: 70%, (1 (66%), 2: 48%, 3 (40%), 4 (42%), 5 (27%)) |
| **Baker et al (2001) ^3^** | No framework mentioned. ‘Adherence’ used as a term for participant engagement. No definition given. | Fidelity | Not measured | Not measured |
|  |  | Engagement | **Procedure & measures:** Self-report questionnaires to physicians (home visit logs) and participants (exercise and food logs) in intervention groups. Dietary logs in control group. Attention control group kept diet logs 3 days of every two weeks for 24 days over 4 months.  **Analysis method:** Two or fewer missing logs = considered 100% adherence. Number of logs returned were counted and divided by 46 for adherence. If no logs were returned it was assumed they were not kept. Descriptive statistics used. | Exercise group mean adherence = 84 ± 27%, with a range of 24-100%.  Control: 65 65 ± 32% with a range of 27-100% |
| **Bailey et al (1990) ^4^** | No framework or definitions provided | Fidelity | Not measured | Not measured |
|  |  | Engagement | **Procedure & measures:** Observation and provider self-report. 10 item observational checklist, developed by project staff (demonstrated as having good reliability and validity) used to assess inhaler use skills**.** Adherence to medications and inhaler use measured by two scales (based on prototype self-report scales described by Morriskey et al, 1986). Adapted for applicability to asthma. Project staff member rated adherence on 3 point scale (poor, fair, excellent). Measured at baseline and 12m follow up in both intervention and control.  **Analysis method:** Not reported – descriptive statistics | Inhaler use – all 10 items correct: baseline 10, 12 month follow up 51.2. Control: 14 baseline, 10 at 12 month follow up. Project staff rating: Intervention 57.5 baseline, 78.3 follow up, control: 50 baseline, 51 12 month follow up.  Inhaler adherence scale – adherent on all 6 items: intervention: 30.4 baseline, 58.3 12month follow up, usual care 59.3 baseline, 61.7 12m follow up. |
| **Binkley et al (2014) ^5^** | No framework mentioned. ‘Dosage’ (caregivers present for entire or part session, or not present. ‘Implementation fidelity’ (adherence) (whether key points were completely/partially/not covered).  No term used for engagement measures (discussed under ‘dosage’) | Fidelity | **Procedure and measures:** Self-report questionnaire (providers) to document level of adherence and video camera observations at post assessment (due to technical problems, provider self-report used.  Fidelity (adherence) measured whether key points were completely covered or partially covered/not covered.  **Analysis method**: Descriptive statistics: Percentage fidelity and counts of participation | Intervention fidelity high, (81%-90% for ¾ components):  Planned action component: 71% of CG completed contract, 71% of CG updated 2 oral health plans, 9% updated 1 oral health plan. Capacity building: Didactic training: 86% all, 9% partial. Observational learning video 86% all, 9% partially. Observational learning demo 76% all, 19% partially. Environmental adaptation 90% all (dental devices), 90% all adaptation strategies. Reinforcement coaching 91% achievement of health goals. |
|  |  | Engagement | **Procedure and measures:** Caregiver participation self-reported by provider (whether caregivers made changes in implementing components and why)**.** Caregiver daily checklist completed by the caregiver each time they provided OH support relating to oral hygiene, dental devices used, rewards and monitoring**.** Data collected 7 days before implementation and 7 days after completing intervention. Dose – caregivers presence for entire session, part session or not at all  **Analysis method:** Frequency data (percentage completion) | 19/21 caregivers completed daily checklist (pre-assessment), 16/21 (post assessment) – 76% retention rate. 20/21 caregivers participated in didactic training and assessment, 18/21 demonstration training and assessment. 16/21 two coaching sessions and assessment (retention 76%). Dose: Planned action: 95% attended briefing, completed baseline. Capacity building: 86% entire, 9% part. Environmental adaptation: 90% entire, 10% part. Reinforcement: 71% had 2 coaching events, 9% had 1. |
| **Black (2014) ^6^** | Based on the NIH treatment fidelity model (Bellg et al., 2004). ‘Delivery of treatment’ (provided as intended; Bellg et al, 2004)  ‘Receipt’ (ability to understand and perform changes; Bellg et al, 2004)  ‘Enactment’; ability to perform learned strategies in life situations; Bellg et al., 2004). | Fidelity | **Procedure and measures:** An Essential Practices Checklist developed for providers to address the model’s 10 principles over the four phases. 120 cases (all cases audited).  **Analysis method:** Continuous review on ongoing basis, percentages. | 100% compliance ensured |
|  |  | Engagement | **Procedure & measures: Treatment receipt**: Verbal verification of understanding and application of changes with the facilitator. Understanding documented in the care plan (audited to ensure compliance). Caregivers instructed to self-monitor behaviour change as a result of the intervention if applicable and programs assessment tools tracked changes at intake and six month intervals.  **Treatment enactment:** Opportunity for the caregiver to share and reflect on lessons learned, extension of the program until facilitator is assured that the client is self-sufficient in specific goals created and verification of enactment of skills assessed upon termination in follow up testing  **Analysis method:** Reviewed on ongoing basis, descriptive (percentages) | Receipt of treatment = documented 100% of the time. Enactment of skills or lessons were documented and verified for 100% of cases |
| **Brug et al (2007) ^7^** | No framework/definitions given - ‘Adherence’/’infidelity’ used | Fidelity | **Procedure and measures:** 2 consultations audiotaped for each dietitian within 1 month after training and second between 5 and 6 months (Intervention and control). Transcripts made of first 15 minutes. Analysed using Motivational interviewing treatment integrity (MITI) code (Moyers et al, 2003) and manual for motivational interviewing skill code (Miller et al, 2003) (blinded). Evaluator scores on MI relevant criteria e.g. proportion of time client talked, empathy, number of reflections.  **Analysis method:** Researcher blinded, one way ANOVA to compare characteristics of control and intervention | First test: MI adherence 1(low)-17(high) – MI dieticians 9.4(5.4) vs controls 6.6(6.1), p difference between groups .15. MI infidelity: 1(low)-17(high) – 9.4(5.4) vs 6.6(6.1), p=0.15. Second test: MI adherence – MI dieticians 6.4(4.0), controls 6.9(4.7), p=.75. Infidelity: 1.6 (3.7), 3.8(4.0), p=0.01 |
|  |  | Engagement | Not measured | Not measured |
| **Butler et al (2012) ^8^** | No framework/definitions. Term used: ‘Uptake of the intervention’ | Fidelity | Not measured | Not measured |
|  |  | Engagement | **Procedure and measures:** Attendance at session/use of online modules. Intervention clinicians had to complete each online learning component before they could access the next  **Analysis method:** Not reported – data count, descriptive | All but one completed 4 parts of online training. Only 10 clinicians did not attend. 76 completed optional booster session at 6 months. 11 clinicians entered new threads but there were 81 posts and 1485 viewings on these posts. |
| **Campbell et al (1998) ^9^** | No framework/definitions | Fidelity | Not measured | Not measured |
|  |  | Engagement | **Procedure and measures:** Attendance at clinic  **Analysis method**: Not reported – percentage | Intervention: 82% attended at least one clinic. Nurse and health visitors spent 915 hours running clinics, 1h and 22m per patient per year. |
| **Chesworth et al (2015) ^10^** | NIH behaviour change consortium (Bellg et al, 2004) framework. ‘Fidelity to treatment delivery’ | Fidelity | **Procedure and measures:** Self-report measures included completion of intervention documentation (three day diaries and daily clinical logs for participants on bladder training and prompted voiding) to measure adherence to the protocol. Clinical logs used by healthcare staff, (nursing staff and healthcare assistants) to undertake and record delivery of the SVP each day (one log per patient per day – document voiding time/best practice components achieved). Sample: Logs sampled from all 8 sites. Stratified sampling. Two time periods of 14 days were sampled from each stratum. Site samples comprising between six and nine 14 day periods.  **Analysis method:** Descriptive quantitative analysis of how well logs performed (% median hours) by 2 researchers. Clinically justifiable responses/10% of data input checked by senior researcher (Thomas, 2015). Missing or incorrect intervals/voiding times not included. | Clinical logs: Returned and analyzed (25% participants) (Intervention: 396 (40 patients), Supported implementation: 320 (31 patients). Percentage of logs according to prompted voiding and bladder training PT 90.4% patients PV, 9.6% BT (intervention). PT 100%, BT 0% (supported implementation). Stage 1: % voiding interval present and correctly documented (Intervention: 83.3%, Supported implementation 89.4%). Stage 2: % both voiding interval and schedule of proposed voiding time present and correctly documented (Intervention 38.9%, Supported implementation: 31.9%). No of clinical logs that achieved both stage 1 and stage 2: (Intervention 154, Implementation: 102). For clinical logs that achieved stage 1 and stage 2: Stage 3: voiding time within 30 minutes (Intervention 54.8%, Implementation 56%), encouragement documented (Intervention 58.4%, Implementation 57.5%), patient asked if they were wet (Intervention 57.9%, implementation 65.9%) |
|  |  | Engagement | **Procedure and measures:** Nurses submitted 3 day diary for participants. Those catheterized not eligible.  **Analysis method:** Diary assessed using filtering system, data input terminated if failed to achieve key quality indicator for stage. Assessment of ‘yes’/’no’ entered into SPSS (from Thomas et al, 2015). Analysed using percentages. | 68.8% diaries received (intervention group), 80.5% supported implementation group). Diaries completed: (Intervention: all, Supported implementation – 5 blank. Entry on each of the 3 days: (Intervention: 52/102 (51%). Supported implementation: 54/82 (65.9%). Entries for ‘time went to the toilet’ on each day (13/102, 12.7%) or three entries in the corresponding ‘leaked’ columns completed (10/102, 9.8%). |
| **Cheung et al (2015) ^11^** | No framework mentioned. ‘Program evaluation’ and ‘treatment implementation’. Discusses Re=AIM model in discussion; (Damschroder et al., 2009) | Fidelity | **Procedure and measures:** Participant (caregiver) self-report at follow up, including satisfactions and benefits, how much they learned and adopted components, extent to which interventionists implemented intervention and whether intervention taught components (0=no, 1=yes)  **Analysis method:** Analysed using percentages | Implementation of components (range 77.0% (thought-records) to 95.9% (stress reduction techniques). (Stress reduction techniques – 194 replies, 95.9% taught. Pleasant events - 191 answers, 90.1% taught. Thought record - 191 replied, 77.0% taught.) |
|  |  | Engagement | **Procedure and measures:** Participant (caregiver) self-report at follow up, including satisfaction/benefits, how much they learned and adopted components, extent to which interventionists implemented intervention, perceived helpfulness (0 not at all, 2 very much) and whether they are currently using (0 not at all, 2 very much)  **Analysis method:** Means and percentages | Cases returned: at least 1 behavioral prescription out of 243- 73.7% returned. 95% of “perceived helpfulness” (stress reduction techniques and pleasant event) = “little” to “very much.”). **Breakdown of techniques:** Stress reduction techniques: perceived helpfulness 185 replies (62.2% little, 34.1% very much). Currently using at least one technique 182 responded (58.8% little, 28% very much). Pleasant events: Perceived helpfulness (171 replies Mean (SD): 1.28 (0.55). 62.6% 1, 32.7% 2. Currently using: 167 replies. Mean (SD): 1.22 (0.58). 61.7% 1, 29.9% r 2. Thought record: Perceived helpfulness: 142 replies. Mean (SD): 1.03 (0.61). 63.4% 1, 19.7% 2. Currently using 143 replies. Mean (SD): 0.86 (0.62). 59.4% 1, 13.3% 2. Management of problem behaviour: Perceived helpfulness (0 = not at all, 1 = little, 2 = very much) 191 replies. Mean (SD): 1.19 (0.58). 63.4% 1, 27.7% 2. Currently using at least 1 technique 190 replies. Mean (SD): 0.97 (0.62), 62.1% 1, 17.4% 2 |
| **Dannhauser et al (2014) ^12^** | No framework mentioned.  Term: Adherence to activities’ (percentage confirmed completion of the offered 28 DSD activities, 36 physical activities and 10 GCSE sessions and 10 supervised ICST sessions. | Fidelity | Not measured | Not measured |
|  |  | Engagement | **Procedure and measures:** Self-report methods and observational methods, including: feedback entries (date, location, comments, 4-point Likert scale) for DSD activities, direct observation for the physical, GCST and ICST activities, data from the logger and entries on the calendar on the participant poster for physical activities. 1) Physical activity: 7 home visits for supervision at set intervals – more frequent visits to facilitate adherence initially and telephone contact to promote adherence. 2) Group based cognitive stimulation training (GCST): supervision and structure and diversifying activities which could reduce engagement.  **Analysis method:** Percentage confirmed completion of the offered: 28 DSD activities, 2) 36 physical activities, 3) 10 GCST sessions and 4) 10 supervised ICST sessions. | Activity adherence rates were high. 63/67. Participants completed more than 50% of activities offered: DSD activities = 83%; Physical activity = 71%; GCST = 83%; ICST = 67%. The mean duration of exercise = 36.3 mins, SD = 8.6 min. The intervention was provided to 10 groups across 5 different locations. Participants received a mean of 4.70 telephone contacts (SD = 2.89, range 1-13). |
| **DeWalt et al (2006) ^13^** | No frameworks or terms mentioned | Fidelity | Not measured | Not measured |
|  |  | Engagement | **Procedure and measures:** Participant self-report: Heart failure self-management behaviour (measured at 12m) assessed by asking patients how often they weighed themselves  **Analysis method:**  Percentage scores, inferential statistics not specified | Intervention: 79%, control: 29%, reported daily weight measurement at 12m (P<0.001) |
| **Driessen et al (2010) ^14^** | Adapted version of the Linnan and Steckler framework. ‘Fidelity’ (‘extent to which steps of PE programme were delivered as intended’). ‘Dose delivered’ (‘perceived implementation of ergonomic measures according to implementers’). ‘Dose received’ (‘perceived implementation of prioritised measures according to workers’ and ‘implementation of measures according to workers’) | Fidelity | **Procedure and measures:** Provider self-report: 4 months after working group meeting, completed a short questionnaire on components of fidelity. Scored using a 10 point scale (very bad to very good). Perceived implementation: yes, partly, not implemented. Measure classified by two researchers independently.  **Analysis method:** Percentage average | 65/81 responses to questionnaire (80%). 34% ergonomic measures implemented, 26% partly implemented, 40% not implemented. Perceived implementation ranged 26%-79% |
|  |  | Engagement | **Procedure and measures:** Intervention receipt: Participant self-report – 6 months follow up questionnaire. Question asking whether implemented – yes/no/don’t know. Workplace implementation yes/no  **Analysis method:** Percentages | Participant level. Dose received: 833 workers completed questionnaire (26% perceived the ergonomic measures as implemented, 36% as partly implemented, 38% as not implemented at the departments.). Compliance to watching movies = 67% in intervention group |
| **Dubbert et al (2002) ^15^** | No framework used. ‘Adherence’ | Fidelity | Not measured | Not measured |
|  |  | Engagement | **Procedure and measures:** Participant self-report (weekly activity diaries) and objective accelerometer (51 participants wore accelerometers.) Participants interviewed about minutes walked for exercise. 7 day PA recall was administered to estimate hours. Validation of walking performed by contacting significant others by telephone to inquire of walking. Nurse blinded to walking diary data and self-report. Received 1$ canteen coupons for each week diary was returned  **Analysis method:** Average monthly percentage adherence as primary self-report activity- number of walking sessions divided by number of sessions prescribed. Missing data = considered non-walking. Displays of accelerometer counts were examined visually. | 50%+ participants initiated walking program meeting study goal. Almost 50% walking at 6m and 40% still recording meeting goal at 10m. Adherence = stable over time. Direct observation confirmed by reports from significant others for 83% of PC, 91% of P and AC and 71% of NC. Accelerometer records matched 72% reported diary walking. |
| **Duff et al (2013) ^16^** | No framework. Process measures (acceptability and utility).  ‘Acceptability’ – no definition  ‘Utility’ – how the EOV was implemented and degree to which the intervention was implemented as intended | Fidelity | **Procedure and measures:** Self-report data collection form (measuring intervention delivery: conducting EOV, number of interruptions, time and location and whether commitment was gained from participant to trial practices)**.** Facilitators self-assessed adherence to elements of the protocol. Two trial visits with self-reflection before  **Analysis method:** Percentage (median and IQR) | Fidelity: 90% (IQR, 87.5–92.5): Amount of times to make contact with participant to arrange an EOV: 2 (IQR, 1–2), Cancellations: 0 (IQR, 0–1), Time spent on each EOV: 63 minutes (IQR, 49–85), Arranging EOV: median, 20 minutes; IQR, 15–20), Customizing the material (median, 10 minutes; IQR, 10–15), Waiting for the participant (median, 20 minutes; IQR, 0–30), Conducting the EOV (median, 11.5 minutes; IQR, 10–15). |
|  |  | Engagement | **Procedure and measures:** Self-report, post intervention surveys (at the end of EOV), containing: questions on effectiveness of EOV at increasing knowledge and addressing concerns, question on effectiveness (5 point scale 1-5), questions on future participation. Facilitator questions measured on a 5 point Likert scale.  **Analysis method:** Percentages | Participant ratings: 84/85 (99%) participants verbally committed to trial new practices. 74% likely/extremely likely to participate in another EOV. 74 (97%) likely/extremely likely to influence clinical practice.  Facilitator ratings: **Participants perceived interest 79 (95%) high/very high).** 31, (41%) very high, 43 (54.2%) high, 4 (4.8%) average, 0 (0) low, 0 (0) very low. **Participation: 79(95%) high/very high.** 34, (41%) very high, 45 (54.2%) high, 4(4.8%) average, 0(0) low, 0 (0) very low. **Comprehension: 71 (85%)**. 45(54.2%) very high, 26(31.3%) high, 12(14.5%) average, 0(0) low, 0(0) very low |
| **Duncan et al (2003) ^17^** | No framework used. ‘Exercise adherence’ – defined as number of exercise sessions completed. ‘Attendance’. | Fidelity | Not measured | Not measured |
|  |  | Engagement | **Procedure and measures:** Exercise adherence (number of exercise sessions completed) measured using exercise diaries. Patient reported attendance 100% agreed with staff recorded attendance.  **Analysis method:** Two tailed independent t tests to determine differences between groups | Group sessions completed: Intervention: 12 weeks: 63.3 (6.4) 24 weeks 59.6(10.6). Control: 12 weeks 59.3 (11.1) 24 weeks 41.2 (9.7)** (P<0.01) |
| **Ettinger et al (1997) ^18^** | No framework mentioned. Terms used: ‘Compliance’ and  ‘attendance’ | Fidelity | Not measured | Not measured |
|  |  | Engagement | **Procedure and measures:** Participants maintained exercise log books (all phases of intervention) + recorded number of prescribed exercise sessions completed and length of session. Attendance determined through provider records. Home based attendance calculated using exercise logs.  **Analysis method:** Compliance (number of sessions), measured in all three groups by dividing by total number of sessions prescribed (3 x a week). If not completed, assumed not exercising. Descriptive – percentages. | Compliance with exercise prescription = 68% in aerobic training group and 70% in resistance training group. Declined during the trial - 85% at 3 months, 70% 9 months, 50% at 18m. No statistical different in compliance between 2 exercise groups. Compliance for the health education program was 91% during first 3 months and 95% for remainder of study. |
| **Farmer et al (2007) ^19^** | No framework used. Terms used: ‘Delivery of intervention’ and ‘adherence to the intervention’ | Fidelity | **Procedure and measures:** Provider (nurses) self-review of taped consultations and external review by sociologist. Prompts built into patient diaries to help patients adhere to intervention. | Not reported |
|  |  | Engagement | **Procedure and measures:** Patient diaries for goals, reviews and activities. Prompts built into patient diaries to help them adhere to intervention  **Analysis method:** Kaplan Meier plot | Use of meter: Ninety nine (67%) less intensive vs 79 (52%) more intensive used metre at least twice a week for 12 months (P=0.012) |
| **French et al (2015) ^20^** | Used Bellg et al (2004) framework. ‘Intervention fidelity’ (whether delivery of the intervention is faithful to protocol | Fidelity | **Procedure and measures:** Workshops audio-recorded and transcribed (apart from workshop content and prompt practice/role play – not captured by recorder). Developed coding guidelines. Observed adherence assessed by coding transcript for BCTs, across facilitators and sessions. Coder recorded whether BCT applied (1) or not applied (0). Some sections double coded (two codes). Coding results discussed and coding frame modified until 80% or more agreement. Random 10% check of coding undertaken by independent researcher, 10% remaining coding undertaken by independent researcher. Facilitator self-reported adherence using a checklist completed at end of each session.  **Analysis method:** Summary statistics to assess observed adherence and to compare self-report and observed adherence. Observed adherence = number of BCTs delivered/number of BCTs planned. Difference in adherence between facilitators assessed using Pearson chi squared test | The observed adherence all workshops was 79% overall, ranging from 33% to 100% per session. BCTs: Persuasive communication: Session 1 21/25 (84%), session 2 18/30, (60%), both sessions: 39/55 (71%). Information provision: Session 1: 14/15 (93%), Session 2: 18/18 (100%), both sessions 32/33 (97%). Provide info on consequences: Session 1: 15/15 (100%), session 2: 4/12 (33%), both sessions: 19/27 (70%). Social comparison. Session 1: 10/10 (100%), session 2: 11/18 (61%), both sessions: 21/28 (75%). Barrier identification: Session 1: 10/10 (100%), session 2 10/12 (83%), both sessions 20/22 (91%). Provide instruction: session 1 0, session 2 14/18 (78%), both sessions 14/18 (78%). Time management: 0, session 2: 5/6 (83%), both sessions 5/6 (83%). Total: session 1: 70/75 (93%), session 2 80/114 (70%), both sessions 150/189 (79%). Sensitivity of self-reported adherence against gold standard (identifying when did occur) = 95% (88-98). Specificity – correctly identifying when a section of a workshop did not occur according to observed adherence = 30% (11-60) |
|  |  | Engagement | Could not be measured | Not measured |
| **Gabbay et al (2013) ^21^** | No framework. Terms used ‘fidelity’/’engagement’ | Fidelity | **Procedure and measures:** MI experts monitored audiotape sessions monthly using the Behaviour change counselling index (BECCI) - reliable and valid tool (Lane et al, 2005)**.** Providers were given feedback based on these evaluations. Nurse practitioner reviewed audio-tapes (weekly reviews, providers and investigators met to review progress biweekly/more frequently if needed)  **Analysis method:** Not reported | Not reported |
|  |  | Engagement | **Procedure and measures:** Monitoring of completion of the study/visits  **Analysis method:** Not reported – descriptive percentages | 197 (85%) completed study. Of these, 75 (32% of intervention group) lost engagement (didn’t see NCM in last 8 months of study). |
| **Goyder et al (2014) ^22^** | Treatment fidelity framework (Bellg et al, 2004). Terms: ‘Fidelity’ (delivered as per protocol), ‘receipt’, ‘enactment’ | Fidelity | **Procedure and measures:** Audio-recorded sessions independently coded using the motivational interviewing treatment integrity (MITI; Moyers et al, 2007) assessment (global ratings of evocation, collaboration, autonomy, direction and empathy). Minimum levels based on levels of competence stated in MITI coding system. MI adherence behaviours (e.g. asking permission, affirming, emphasising personal control), MI non-adherent behaviours (e.g. advising, confronting, directing), open compared with closed questions and simple and complex reflections.  **Analysis method:** Counts of MI adherent and MI non adherent behaviours made across each RA and domain - descriptive statistics | 73% (136/186) received the intervention as per protocol [77% (71/92) in the mini booster arm and 69% (65/94) in the full booster arm]. % MI adherent (means): RA1 88%, RA2: 50%, RA3: 100%, RA4: 100%. RA5 100%, RA6: 100%. Motivational interviewing treatment integrity ratings (proficiency is 3.5, competency is 4) means: Evocation: Ra1 3.5, RA2 2.5, RA: 4, RA4: 3, RA5: 4, RA 6: 3. Collaboration: RA1 3.5, RA2 3.5, RA3 3.5, RA4, 3.5, RA5: 3, RA6, 3. Autonomy: RA1: 3.5, RA2: 2, RA3: 3, RA4, 3.5, RA5 4, RA6 3 . Direction: RA1: 4, RA2: 5, RA3: 5, RA4: 4.5, RA5, 4, RA6, 2. Empathy: RA1 4.5, RA2, 3, RA3: 3, RA4: 3.5, RA5: 4, RA6: 4. Average global rating: RA1: 3.5, RA2, 3, RA3: 3.5, RA4 3.5, rA5 3.6, RA6 3. % Complex reflections: RA1: 45, RA2: 18, RA3 18, RA4 34, RA5 38 RA6 40. % Open questions: RA1 40, RA2 36, RA3 45, RA4 41, RA5 30, RA6 33. . Reflection to question ratio. RA1 2.6, RA2 1.2, RA3 1.2, RA4 1.2, RA5 1.8, RA6, 1.6 |
|  |  | Engagement | Mentions measuring receipt and enactment | N/A |
| **Griffin et al (2010) ^23^** | No framework used. ‘Program delivery’ and ‘fidelity’ (extent to which the intervention was delivered as planned) (Saunders et al, 2005). ‘Participant level engagement’ (dose/attendance/participation) | Fidelity | **Procedure and measures:** Program staff completed data entry worksheets corresponding to essential elements (group size, deliver all 20 sessions, completion of check-in activity, incentives provided, stage of readiness for change, learning activity implementation, modifications made to session, home assignments given). Dose and fidelity captured through electronic reporting system. Reviewed for completeness  **Analysis method:** Percentage of sessions delivered as intended (out of 20), as opposed to combined with other sessions. Percentage of sessions that included a check in activity. | An overall high level of implementation fidelity was observed. Group sizes ranged from 4-33, 6% of sessions were combined. **Components:** Check In completed % (all years) 95.7% (88.9-99.1), Incentives provided % of sessions, all years – 51.7% (14.70099.6), Sessions staged: 4.1 (1.1) (3.8-4.3), Learning activities per session 3.4 (1.6) (3.3-3.6), Learning modification ^ of sessions 15.8 (1.7-26.6), Home assignments given per session 4.2 (2.0) (3.3-4.6) |
|  |  | Engagement | **Procedure and measures:** Participant self-report. Two measures of participant engagement: completion of home assignments (20 sessions) and participation in self-monitoring activities – percentage of participants who tracked thoughts, types of PA, days and minutes and steps.  **Analysis method:** Percentages | Attendance: average attendance rate of 65%. Homework completion % of participants: 72.2% (52.9-83.3%). Tracking PA (self-monitoring % participants 38.3 (8.9-62.1). Tracking activity by type of activity % of participants 47.9 (8.9-64.5). Tracking steps, % of participants 62.8% (34.6-78.1). Tracking activities by days and minutes of activity % of participants 27.1 (6.7-50.2). Any type of tracking % across sites 67.6 (28.2- 80.2) |
| **Grubbs et al (2015) ^24^** | No framework. ‘Dose of CBT’ and ‘Engagement’ – homework adherence and commitment to CBT | Fidelity | **Procedure and measures:** Data entered by the care manager following each clinical encounter for those randomised. CBT dose measured in the intervention group (number of sessions, participation in relapse prevention calls, interruption in treatment, number of CBT modules completed and total number of exposure exercises completed)  **Analysis method:** Means and percentages/odds ratios (descriptive plus inferential) | Amount receiving services (all these are labelled ‘dose’): 87% received CBT, 33% received CBT alone, 54% received CBT and pharmacotherapy, 9% received pharmacotherapy alone, 4% received no service. Relapse prevention *received relapse prevention phone calls following completion = 1.38 (odds ratio). CALM CBT modules * mean number of CBT modules completed during course of treatment = 1.17 odds ratio. CALM CBT exposures *total number of exposure modules completed during course of treatment = 2.44 odds ratio. Number of CBT sessions 1.18 (odds ratio). Interrupted treatment (odds ratio) 1.04 |
|  |  | Engagement | **Procedure and measures:** Engagement was measured in the intervention group by clinicians at the completion of each session and included homework adherence (4-point scale; 1 missed most, 4 missed none) and commitment to CBT (0–10 point scale; 1 none, 10 complete)  **Analysis method:** Means and percentages/odds ratios (descriptive plus inferential) | Attending psychotherapy sessions: Women attended a greater number of CBT psychotherapy sessions than men, (7.3 vs. 6.5; O.R. = 1.18, p = .01). Mean number of sessions for each group were within the recommended range (6–8 sessions). Relapse prevention *received relapse prevention phone calls following completion = 1.38 (odds ratio). CALM CBT modules * mean number of CBT modules completed during course of treatment = 1.17 odds ratio. Greater for women (OR=2.44 p=.01). CALM CBT exposures *total number of exposure modules completed during course of treatment = 2.44 odds ratio. Homework adherence: Odds ratio = .93. CBT anxiety commitment: Odds ratio 1.26. The clinician-rated measure of commitment (O.R. = 1.26, p = .04) was significantly higher for women |
| **Hankonen et al (2014) ^25^** | No framework used. ‘Enactment’ – use of BCTs in daily life | Fidelity | Not measured | Not measured |
|  |  | Engagement | **Procedure and measures:** Participants in the intervention group completed a questionnaire (previously piloted), assessing use of eight BCTs (increasing physical activity) and eight BCTs (eating lower fat diet) in the past 11 months (e.g., goal setting, action planning, self-monitoring. Used a binary scale (yes/no).  **Analysis method:** Examined if BCTs used differed across ages and genders. BCT use categorised into three groups: 1) used all 16 BCTs, 2) 11-15 BCTs, 3) 0-10 BCTs. ANOVA and ANCOVA used. | Out of 239 intervention participants, 210 (87.9 %) participants provided valid data on all variables at 1 year. 35.7% participants all 16 BCTs. BCT use ranged from 61.5% to 88.3%. 40.5% participants used all 8 PA BCTs and 46.2% reported all 8 diet BCTs.  Individual BCTs: Goal setting: PA 34 (15.40%) no, 187 (84.6% yes), diet 26 (11.70% no, 196 (88.30% yes). Action planning: PA 54 (24.50% no, 166 (75.5% no. Diet 43 (19.5% no, 178 80.5% yes). Using prompts/reminders PA 84 38.5% no, 134 61.5% yes. Diet, 73 33.3% no, 146 66.7% yes. Motivating oneself 44 (20%) no, 176 80% yes. Diet 34 15.5% no, 185 84.5% yes. Social support PA 81 (36.8%) no, 139 63.2% yes. Diet, 58, 26.4% no, 162 73.6% yes. Self-monitoring PA 64 (29.1% no 156 70.90% yes). Diet 70 31.70% no, 151 68.3% yes. Goal review 69 31.20% no, 152 68.8% yes, Diet 67 30.3% no, 154 69.70% yes. Preparing for/dealing with setbacks 85 38.5% no, 136 61.5% yes. Diet: 73 33% no, 148 67% yes |
| **Hardeman et al (2008) ^26^** | Bellg et al (2004). ‘delivery’/’fidelity’/’adherence’ | Fidelity | **Procedure and measures:** Assessed fidelity using a protocol which specified various behaviours against transcripts of tape recorded sessions (0, not applied, 1 applied). Coding frame developed for each session. Two researchers classified, 2 independent raters validated (72% agreement). Observed adherence = number of component behaviours applied divided by number of behaviours**.** Coding frame piloted (discussed disagreements until at least 75%) using 22 transcripts. Median interrater agreement 82% in remaining 19 rounds (78-91%)**.** Independent rater assessed 108 transcripts (purposively sampled), second rater assessed sessions 1 and 4 for all participants (54) (interrater agreement over 75% for 76/86 (88%) behaviours). Median agreement = 86% - similar for session 1 and four and intra-class correlation was high at 0.96 for all behaviours across sessions.  Facilitators reported delivery of nine techniques after each session (0 not covered, 1 covered).  **Analysis method:** Variability in adherence assessed using Page test. Differences in adherence to each technique – t test. | Mean adherence to individual techniques across all sessions ranged from 25% (generalising skills to other behaviours, e.g., healthy eating) to 66% (summarising, defining the agenda), with an **overall mean of 45%**.  Observed components (across all sessions): Building support 43.3% (24.2). Strengthening motivation 50.8% (19.8). Goal setting 62.2% (18.6). Action planning 50.3%(17.4). Self-monitoring 49.7% (18.0). Using rewards 54.1% (22.8). Goal review 42.8% (14.9). Using prompts 33.9% (15.2). Preventing relapse 26% (18.5). Building habits 36.1% (20.6). Generalising skills to other behaviours 24.6% (23.3). Eliciting questions 51.3% (26.7). Summarising, defining agenda 66.2% (24.7). Providing information 50% (19.6). Total 44.8 (15.9). Use of techniques by facilitators varied considerably across participants (18–71%), with a median (IQR) of 44% (35–62%). Differences between observed and facilitator-reported adherence. 44% for observed adherence and 100% facilitator reported adherence (97-100%) |
|  |  | Engagement | Not measured | Not measured |
| **Harting et al (2004) ^27^** | No framework used. ‘Quality of health counselling’ | Fidelity | **Procedure and measures:** Used video-recordings to rate fidelity using an observation list (acceptable internal consistency) which covers regulating skills and therapeutic alliance (Part 1: therapeutic alliance, part 2: general interview skills, part 3: exploration of aspects of behaviour change, part 4: basic counselling attitude). Each part = several subscales, all measured by one+ items and overall mark. Scored on one of two four point rating scales: ‘unsatisfactory, doubtful, satisfactory, good’ and ‘not at all, hardly, slightly, considerably, strongly’. Also not applicable. Sufficient overall score = 66%**.** Sample: 64 video recordings. Not randomly selected. Recordings made on previously defined days.  **Analysis method:** All 64 assessed by three independent observers. Reliability assessed using weighted kappa and multi-rater kappa. Descriptive statistics to quantify fidelity | Overall quality of counselling: 2.85 (sufficient score = 3 or 66%)  Regulating skills – 2.65, Interview skills – 2.85, Steps of behavior change = exploration: 2.79, Steps of behavior change Intervention = 2.66, Steps of behavior change basic counselling attitude = 3.15, Health advisors basic counselling attitude (3.15 – satisfactory), Interview skills (2.85) |
|  |  | Engagement | Not reported | On average, participants attended 2.31 counselling sessions which took a total of 83 minutes. |
| **Hermens et al (2001) ^28^** | No framework. Use of term ‘exposure’ | Fidelity | **Procedure and measures:** Self-report questions about exposure to programme elements: (a) informed about the programme (yes/no); (b) type of contact with project staff (outreach visitor or district GP coordinator): contact through CME or small group education for GPs (yes/no), contact through CME or small group education for practice assistants (yes/no), contact through one or more practice visits (yes/no), and number of practice visits; (c) use of the specific software (yes/no).  **Analysis method:** Percentages | 94% informed about national prevention programme, 70% Contact with outreach visitor or district GP, 30% CME or small group education for GPs, 30% CME or small group education for practice assistants. For practice visits that had contact with an outreach visit (40%), mean practice visits were 2 (1-13) |
|  |  | Engagement | Not reported | Facilitating software used by 474 practices (48%) either in full or in part. |
| **Holtrop et al (2015) ^29^** | RE-AIM framework. Terms: ‘adoption’/’implementation’ | Fidelity | **Procedure and measures:** Observation and interview ratings by multiple independent raters. Implementation rated as excellent, good, fair or poor. Research team independently rated each practice on how well and often they used processes on a 4 point scale (4 used well, 3 used well but not often, 2 a mix of used well and not well and 1 not used or not used well). Discrepancies resolved and consensus agreed. Ratings given from review of interview data (conducted by two researchers, in 5 practices) about knowing how to use program, reported use, meaning, value, enthusiasm, support + observations of practices. Field notes collected using a structured observation template to describe: physical environment, practice personnel and culture and patient population + RAs completed a one page summary report describing key findings  **Analysis method:** 5 researchers developed coding guidelines, interviews coded, interrater reliability evaluated, met to reconcile scores and resolve discrepancies. Descriptive | Practice A: Good, B: Good, C: Fair, D: Fair, E: Poor. (Qualitatively derived ratings). |
|  |  | Engagement | **Procedure and measures:** Adoption: The number of providers referring to CM. Maintenance: Patient follow up completion rates  **Analysis method**: Percentage and number of patients. | Adoption (proportion of providers referring 5 or more patients to care manager: A: 3/3, B: 6/6, C: 7/8, D: 2/4, E: 6/8. Maintenance: (6 month follow up rate of patients with the care manager for the scheduled assessment: A: 70.3%, B: 52.1%, C 40%, D 48%, E 38%. |
| **Hunt et al (2001) ^30^** | No framework or terms/definitions given – discussed as ‘process variables’ | Fidelity | **Procedure and measures:** Participant self-report, at end of telephone interview. Participants asked: whether they saw provider as scheduled, whether they spoke about nutrition, whether they reviewed the Eat smart recommendations and how many minutes they spent talking about nutrition. Provider self-report (n=28; 75% response rate) at end of intervention. Providers given a 6 item survey. Asked how often talked with participants about benefits of healthy eating, when provided nutrition information, how often considered Eat smart recommendations, time taken to discuss. Response options: Never, most of time, often, always and do not remember  **Analysis method**: Percentages | 71% discussed diet health relationship/tailored recommendations with providers, 57% reported discussing complete diet health endorsement, 71% providers reported acknowledging the diet health relationship often or always, 62% said they gave eat smart recommendations often or always. |
|  |  | Engagement | **Procedure and measures:** Participant self-report, at end of telephone interview. Participants asked: whether they saw provider as scheduled, whether they spoke about nutrition, whether they reviewed the Eat smart recommendations and how many minutes they spent talking about nutrition. Response options: Never, most of time, often, always and do not remember. Invited 230 patients to participate, 217 completed first interview, 183 completed second interview.  **Analysis method:** Percentages | 89% reported setting goals, 57% reported reaching first goal, 74% reported fruit and vegetable consumption being emphasized. 16% reported primary focus as red/processed meats, 16% low fat dairy products and 10% whole fat dairy products. 7/230 participants referred to registered dietitian. |
| **Yu-Yahiro et al (2009) ^31^** | Resnick et al (2005). ‘Delivery of treatment’ (delivered as intended) | Fidelity | **Procedure and measures:** 70 randomly selected home observations by 2 observers (nurse researcher + exercise physiologist) measured using a checklist during the entire study intervention period across all intervention groups. Participant exercise log books monitored to assess fidelity. Feedback to the interventionist was provided and ranged from specific exercise intervention techniques to reminders to use the exercise calendars, review the exercise booklets, or incorporate verbal encouragement. Quarterly treatment fidelity visits (by 2 investigators) for each provider.  **Analysis method:** Percentages | Adherence to the delivery: 91%. 92% of participant’s demonstrated evidence that they received the intended intervention during these observations. |
|  |  | Engagement | Not reported | Average number of visits (intervention) : 36.2 (65%). If one trainer visit, average number: 44 (78.5%) |
| **Jansink et al (2013) ^32^** | No framework. ‘Exposure’ | Fidelity | Not measured | Not measured |
|  |  | Engagement | **Procedure and measures:** Exposure of nurses to intervention measured by recording attendance at training sessions. Asked nurses if they used the instruction chart. Recorded number of nurses who received three telephone follow up calls and participation in meeting.  **Analysis method:** Percentages | 93% nurses attended at least three of 4 sessions, 74% discussed social maps and diabetes protocols. Most nurses used chart during or after consultations. |
| **Keith et al (2010) ^33^** | ‘Fidelity of implementation’ (‘consistency and quality of targeted organisational members use of the specific innovation’ (Klein & Sorra, 1996), ‘Satisfaction’ – enthusiasm, ‘Quality’ – competence and knowledge, ‘Consistency’ – frequency used the intervention based on guidelines | Fidelity | **Procedure and measures:** Qualitative rating based on participant interviews with 18 clinicians.   1. Delineate 8 components of intervention. 2. Rate intervention components (a) satisfaction, perceptions of quality, consistency of use of components), b) matrix for each participants, c) two authors code text for 5 transcripts – 25% of data, d) codebook to specify rules. e) One author coded and assigned ratings. f) 4 authors reviewed and agreed on final categories. g) Reviewed and discussed matrix. h) FOI scale amended to 5 category scale to include high and low compliance. i) differences in opinions discussed).   Organizational members’ commitment to use was rated on a scale consisting of five categories: (1=nonuse, 5=committed use). Sample: Purposive sampling  **Analysis method:** Assessed at organizational member level, overall medical centre (aggregated) - descriptive | Average ratings: A: 3.4, B: 3.6, C:3.5, D:, 3.0 (1 = nonuse, 2 = low compliance, 3 = compliant, 4 = high compliance 5= committed)  FOI ratings for components: Availability of nurse practitioner case manager (A 4, B 4, C 3, D 3). Collaboration between primary care providers and NP case managers (A 3, B 4, C 3, D 3). Coordination between primary care centers and inpatient centers (A- , B 4 , C 4 , D 3). Provision of video conferencing sessions (A 3, B 5, C 4, D 4). Provision of telemedicine technology (A 1, B 3, C 1, D 1). Provision of patient education documentation (A 3, B –, C 5, D -). Provision of laptop computers (A 5, B 3, C 3, D 3). Provision of case manager training (A 5, B 2, C 5, D 2) |
|  |  | Engagement | Not measured | Not measured |
| **Lawrence et al (2014) ^34^** | No framework | Fidelity | **Procedure and measures:** Observed how many times trainers modelled the skills during training (Used Flanders Interaction Analysis technique – every 10 seconds, researchers recorded whether trainers were asking exploratory questions (skill 2), modelling the skills, speaking using other forms of question or instruction and speaking or undertaking an activity. Not possible to record use of reflection (skill 3). Recorded who did most of the talking and time spent asking open questions. SMARTER planning recorded (skill 5). Observed all trainees and attended as many sessions as possible. Telephone call fidelity assessed by checking questions asked against those on script.  **Analysis method:** Percentage of time trainers were modelling skills and trainees were undertaking activities were calculated as measures of fidelity to the manual. Skill 2 was assessed through making a tally of the questions asked and categorising them into either open discovery questions or other responses. | 76% of training sessions spent doing activities/speaking. Open questions used nearly 1/3 of time. |
|  |  | Engagement | Not measured | Not measured |
| **Lazovich et al (2000) ^35^** | No framework – just referred to as ‘process evaluation’ | Fidelity | **Procedure and measures:** Flexible about implementation to minimise disruption. Intervention physicians recorded on a physician script whether the intervention was delivered. Participants asked about delivery 3 months later.  **Analysis method:** Descriptive statistics. A Mantel-Haenzel X2 statistic was used to evaluate differences in use according to who delivered the booklet and the amount of time spent discussing it. | Provider report: 92.5% received intervention.  Participant interview: 96% received booklet, 50% from doctor, 27% from nurse/other clinic member, 18% researcher assistant, rest in mail. 62% reported the intervention lasting 2 mins or less. |
|  |  | Engagement | **Procedure and measures:** Participants interviewed 3 months later. Intervention: Asked about receipt of booklet, when visited doctor, job title of provider, amount of time discussing diet change, use of booklet (self-reported reading of one+ section of booklet by 3months). Asked control participants at 3m interview if they received any written materials on diet.  **Analysis method:** Descriptive statistics. | 92.8% said they had read some or all of the booklet. Participants were more likely to read booklet if they discussed between 1-3mins. |
| **Lobb et al (2004) ^36^** | No framework. ‘Dose of intervention delivered’, ‘Fidelity to intervention protocol’, ‘Dose of intervention received’ | Fidelity | **Procedure and measures:** Self-report on computerised process tracking system**.** Dose delivered (length and completion of activity on laptop after activity). Extent: How many intervention activities per participant, average number of telephone calls completed and average number of tailored materials mailed. Fidelity: Proportion of participants who received key intervention components as planned, ICS on the same day as the clinician visit, physical activity clearance on the same day as the ICS, ICS at the health centres, and mean length of initial counselling session, telephone sessions and proportion of call attempts. Protocol – scored a 1 if completed or a 0 if not completed. Index of six = all activities complete. Certified motivational counselling trainer reviewed 10% of audio-recorded sessions with health advisors to assure compliance and adherence to protocol.  **Analysis method:** Percentages | Implementation score: 5.4 (0-6 activities). The overall implementation score was 5.4 (0–6 activities). Counselling session delivery: 96% participants. All four telephone counselling sessions: 81% 79% participants received clinician endorsement on same day as ICS. 79% received PA clearance on same day as ICS. 86% completed at the health centre. 67% index of 6 for protocol completion – majority completed all activities, 90% >5. Dose: Mean length = 25 min. |
|  |  | Engagement | **Procedure and measures:** Self-report (for tailored materials only) 8 month follow up survey to measure patients’ receipt of tailored materials. Asked how many of the materials they read, how helpful they were in setting personal goals for changing habits. Scored using ‘yes/no/don’t recall’, ‘most, all, some, none’, ‘very helpful, neither helpful nor unhelpful, very unhelpful’  **Analysis method:** Percentages | 842/1088 participants (77%) received the clinician endorsement). A total of 967 (89%) intervention participants responded to the follow-up survey. 876 (91%) reported that they received tailored materials from Healthy Directions-HC. 76% reported reading most or all of the materials. |
| **Matei et al (2015) ^37^** | No framework. Term: ‘adherence to tips’ | Fidelity | Not measured | Not measured |
|  |  | Engagement | **Procedure and measures:** Participant self-report using 7 day tick sheets to record adherence to tips. For one tip, which recommended setting a manageable walking target, participants were asked to record their daily target and whether it had been achieved.  **Analysis method:** Weekly adherence: summing number of ticks that week divided by 7. Mean total adherence summing all ticks for each tip and dividing by 49 (7days x 7 weeks). Global mean adherence summing mean total adherence to each of 16 tips and divided by 16. All rates were x 100 to express percentages | 92% returned at least 8 tick sheets (both samples), one didn’t return anything (sample 1,2) one returned 5 (sample 2). Global mean adherence: 40.48% (sample 1) and 57.86% (sample 2).  Sample 1: Mean total adherence rates were highest for Tip 2 (‘make ad breaks active’; 60.85 %, range 45.45 % to 67.53 %) and lowest for Tip 5 (‘tiptoe through the queue’; 14.84 %, mean per-week adherence range 6.49 % to 35.06 %). Sample 2: Mean total adherence rates were lowest for Tip 5 (‘tiptoe through the queue’; 38.97 %, range 31.97 % to 42.21 %), and highest for Tip 1 (‘leave the house daily’; 81.63 %, range 71.43 % to 85.71 %). Sample 1: Highest adherence observed between weeks 2 and 4, with the exception of Tips 9c (‘toe rises’; weeks 2 and 6) and 9 g (‘lift a tin of food in each hand’; week 7). Lowest adherence for all tips was observed at weeks 7 or 8. Sample 2: Highest adherence was typically observed between weeks 2 and 4, and lowest adherence between weeks 5 and 8, most typically at week 8. Sample 1: Mean adherence was above 50 % for five of the sixteen tips (Tips 1, 2, 9a, 9b, 10), indicating these were typically more often enacted than not. Sample 2: Mean adherence above 50% for eleven tips (Tips 1, 2, 3, 4, 7, 8, 9a, 9b, 9c, 9e, 10). |
| **McCarthy et al (2015) ^38^** | No framework. Implementation of intervention according to protocol (including fidelity, dose and context), ‘Engagement’ | Fidelity | **Procedure and measures:** All 20 exercise counselling sessions audiotaped. Four (20%) sent to an independent expert in MI for review and assessment. Assessed for: average of spirit global, reflection to question ratio, percent open questions, percent complex reflections, percent MI adherent, scored each session). 90% reflects beginning proficiency. Dose: examining the quantity or amount of intervention delivered to participants. This consisted of three components: the exercise counseling session, telephone follow-up, and use of the daily diary. Participation in each component was calculated  **Analysis method:** Percentages | 100% audiotaped. All essential elements covered as planned. Adherence to principles of MI was 40–50% in subject 4 and 5 (low adherence). Increased to 100% and 80% in subject 16 and 20. (168/180 calls with collected data (93% of calls successfully made/step data collected). Global spirit clinician rating: 2.3 and 2.7 (two interviews) – lack of proficiency compared to 3.5 average. Adherence to MI improved to 80–100%, but the other ratings are not all consistently higher. |
|  |  | Engagement | **Procedure and measures:** Adherence to daily dairy was tallied for each of four activities (daily step- counts, body weight, use of the hand weights, Borg scale).  **Analysis method:** The total number of actual recorded data for each activity was divided by the number of potential diary recordings (15 participants ×84 days = 1260). This resulted in the percent adherence in each activity. | Step count recorded: 801/1260 = 64% of days. Borg scale: 628/1260 = 50% of day. Daily weight: 651/1260 = 52% of days. Hand weight use: 400/1260 = 32% of days |
| **McCurry et al (2012) ^39^** | NIH Behaviour change consortium (Bellg et al, 2004). Data on: Treatment delivery (SETP implemented in a standardised way across settings). Receipt (whether SEP concepts and components were understood by participating staff caregivers). Enactment (whether staff caregivers followed treatment recommendations with their residents) | Fidelity | **Procedure and measures:** Self-report checklist completed after each session indicating which of the 13 SEP topics had been covered (keeping a sleep log, assigned readings, sleep changes in dementia, behavioural treatment rationale, developing a sleep plan, monitoring problematic sleep behaviours, identifying activators for problem behaviours, changing caregiver responses to behaviours, implementing sleep plans, improving communication with residents effects of light, daytime activity and pleasant events on sleep and mood). All sessions audio-recorded and reviewed by PI who provided feedback.  **Analysis method:** Not reported – descriptive statistics/percentages | Sleep Education Program was delivered as intended in active treatment. All topics covered, coverage varied. Sleep log monitoring and resident sleep plans discussed every visit. Relationship between sleep and dementia, monitoring behaviours and activators discussed in 60Other components delivered in first two or last two sessions. Assigned as homework: Sleeping schedule 90% (89%-92%), Reduced napping: 89% (83%-92%), Increased exercise 64% (61-67%), Light changes 48% (40-54%), Other 26% (23-32%). Sessions averaged 41.5 minutes (range: 6–80 minutes). |
|  |  | Engagement | **Procedure and measures: Receipt:** Provider self-report (Staff caregiver attendance at SEP sessions and clinical impressions (staff interest, understanding, willingness to make changes, conduciveness of AFH environment) rated by trainer after session. Recorded whether staff-caregivers able to identify sleep related target behaviours for change, potential activators and consequences and develop behavioural sleep plans for upcoming week. Rated on 0-3 scale (0 not at all, 3 fully). Willingness, interest and supportiveness on a 1-5 scale (1=not at all, 5 extremely)  **Enactment –** Provider reviewed homework at every SEP session, rated compliance (attempted, not attempted), assisted staff caregivers in problem solving. Rated success implementing treatment recommendations  **Analysis method:** Descriptive and inferential stats – (inferential stats not specified) | **Attendance at sessions:** 27 (73%) caregivers participated in all four individual sessions (mean: 3.6 sessions, range: 1–4). **Compliance with actigraph recorders:** 88% residents provided 6 or more days of actigraph data at each of the three sampling points. **Receipt:** 7% fully understood concepts session 1, 67% fully understood in session 4 (p<0.0001). 62% staff intervention condition extremely interested, 48% extremely willing to participate, 69% supportive environment. **Enactment:** 16% established ABC plans in session 2, vs 88% session 4 (p<0.0001). 13% scheduled increased pleasant events in session 2 vs 92% session 4 (p<0.0001). **Homework attempted:** Sleeping schedule changes: 76% (71-87%), Reduced napping 66% (57%-73%), Increased exercise 45% (39%-56%), Light changes 41% (39%-45%), Other environmental changes 23% (21%-24%) |
| **McGillion et al (2008) ^40^** | No framework. ‘Intervention delivery’ /process evaluation (‘attendance’) | Fidelity | **Procedure and measures:** All sessions audio-taped. Random sample (10%) externally audited to ensure standard intervention delivery. | Not reported |
|  |  | Engagement | **Procedure and measures:** An attendance record was kept to track the number of CASMP sessions attended by the treatment group participants.  **Analysis method**: Percentages | 93% attended all six program session, 7% attended three or more. Average number of sessions attended: 5.8 |
| **McNamara et al (2015) ^41^** | No framework. ‘Intervention fidelity’. 1) Appropriateness and suitability of intervention structure, retention of patients, time taken to deliver intervention. 2) Appropriate targeting and delivery of intervention (Recruitment, recommendation of goals addressing risk factors, patient agreement, strategies to address risk factors, identification of barriers/enablers. 3) Perceived success of behaviour strategies. 4) Perceived competence | Fidelity | **Procedure and measures:** Community pharmacists documented issues about each session to monitor nature of counselling**.** Goals and strategies only included if related to health behaviours. Perceived success of behaviour change strategies. Self-assessed perceived competence by pharmacists to deliver intervention after session 5.   - Pharmacist perceived need for further support - Perceived success: yes/no – yes = success - Perceived competence – yes/no after session 5.   **Analysis method:** Percentages | Pharmacists felt competent to deliver intervention 94% patients. **Weight loss: 53/63, 84**% goal recommended for adoption, % with a documented strategy to address goal (n-64). Dietary 58 (91%), alcohol, 11 (17%), physical activity 56 (88%), other weight 15(23%). % of participants who agree with participants to pursue a goal relating to this 53 (100). % with a related written strategy. Dietary 47/53 (89%), alcohol 10/52 (18%), physical activity 48/53 (91%), other weight 15/53 (28%). **Increase physical activity:** % with a goal recommended for adoption 47/63 (75%). % with a documented strategy to address goal (n-64). 56 (88%). % of participants who agree with participants to pursue a goal relating to this 44 (94%). % with a related written strategy. 40 (91%). **Improve diet:** % with a goal recommended for adoption 52/62 (84%). % with a documented strategy to address goal (n-64). 58 (91%). % of participants who agree with participants to pursue a goal relating to this 51 (98%). % with a related written strategy. 46 (90%). **Reduce salt intake:** % with a goal recommended for adoption 8/62 (13%). % with a documented strategy to address goal (n-64). 58 (91%). % of participants who agree with participants to pursue a goal relating to this 8 (100%). % with a related written strategy. 8 (100%). **Improve medication adherence:** % with a goal recommended for adoption 11/62. % with a documented strategy to address goal (n-64). 17 (27%). % of participants who agree with participants to pursue a goal relating to this 11 (100). % with a related written strategy. 6 (55). **Lower risky alcohol intake:** % with a goal recommended for adoption 8/62. % with a documented strategy to address goal (n-64). 11(17%). % of participants who agree with participants to pursue a goal relating to this 8(100). % with a related written strategy. 5/7 (72%). **Quit smoking:** % with a goal recommended for adoption 6/62. % with a documented strategy to address goal (n-64). 3 (5). % of participants who agree with participants to pursue a goal relating to this 5. % with a related written strategy. D3 (60)  Dose: Session 1: mean of 34 min, Session 2: 22 min. Further sessions: 15– 20 min for subsequent sessions. |
|  |  | Engagement | **Procedure and measures:** Community pharmacists documented issues about each session to monitor the nature of counselling provided (patient agreement with suggested goals, progress towards behavioural goals and barriers experienced, and agreed changes to goals). In sessions 2–5, pharmacists were asked to rate, as a binary outcome (yes/no), if any success had been achieved for each agreed patient strategy noted in the previous session. At the end of the intervention, pharmacists were asked to document those areas where they felt patients had made major achievements,  **Analysis method:** Percentages | Completing sessions: Session 1: 66 (96%), Session 2: 62 (90%), Session 3 59 (86%), Session 4: 57 (83%), Session 5: 56 (81%) |
| **Metzelthin et al (2013) ^42^** | Framework: Baranowski and Stables (reach, dose delivered, fidelity, dose received (exposure and satisfaction) and barriers (Baranowski and Stables, 2000; Linnan and Steckler, 2002; Saunders et al, 2005). ‘Fidelity of delivery’ (delivered in intended manner). ‘Exposure’/’adherence’ (extent of active engagement in and receptiveness to care approach) | Fidelity | **Procedure and measures:** Practice nurse evaluation form (self-report) and qualitative interviews. Dose: Logbooks contained information about amount of care.  **Analysis method:** Data analysed by three members of research group. Qualitative and quantitative data integrated to cross validate findings. Percentages | Step 2: Assessment by practice nurse: 98.9% 186, Additional assessment GP (43.6%) 82 occupational therapist, 19.1% 28Physiotherapist 14.9% 28, other 9% 17. Step 3: Analysis and preliminary treatment plan: Practice nurse 12.2, %, bilateral meeting (nurse and GP) 64.4%, extended team meeting 22.3%. Step 4: analysis and preliminary treatment plan Practice nurse 23 (12.2%), Bilateral meeting 121 (64.4%), Extended team meting 42 (22.3%). Step 5: Agreement on treatment plan: practice nurse 51.6%.97  Step 6: Executing the treatment plan - use of toolbox parts: Meaningful activities (32: 17.0%), adapting the environment activities or skills 48 (25.5%), social network and social activities 27 (14.4%), daily physical activity 36 (19.1%), stimulate health 41 (21.8%), other interventions 27 (14.4%). Step 6: Evaluation and follow up: Nurse and older person 50%, bilateral meeting 18.6%, extended meeting 28.7% Dose: Log books filled in for 188 people (6 logbooks missing).82 (43.6%) seen by their GP during assessment phase |
|  |  | Engagement | **Procedure and measures:** Practice nurse evaluation form – asked to judge the frailty of the participants (reach), ability to understand the goal and working method of care approach and adherence to commitment/exposure (Baranowski and Stables, 2000; Linnan and Steckler, 2002; Saunders et al., 2005).  **Analysis method:** Percentages | 75.8% understood the goal and procedure. Adherence assessed by nurse as very good (72, 48.3%), sufficient 46(30.9%), poor 30(20.1%), 11 (7.4%) not applicable) |
| **OBarzanek et al (2007) ^43^** | No framework. ‘Adherence’ | Fidelity | Not measured | Not measured |
|  |  | Engagement | **Procedure and measures:** Self-report using the DASH adherence index. The DASH adherence index is a simple average of sub-indices measuring daily intake of dairy servings, fruits and vegetables servings and percent saturated fat. Score of 0-1 = within target range. 1+ indicates intake better than target range. Less than 0 = worse than target range. Validity tested.  **Analysis method:** Descriptive and parallel analyses – multivariate models | (score between 0 and 1 indicates adherence)  Advice only baseline -1.1 (0.7), 6 month change 0.2(0.8)*, 18 month change 0.1(0.8*). Established intervention mean (sd) baseline -1.1 (0.7), 6 months 0.2(0.8) ***, 18 month change 0.1(0.8)**. Established and DASH mean -1.0 (0.7), 6 month change 1.1 (0.9)***, 18 month change 0.9(0.9)*** |
| **Ockene et al (2012) ^44^** | No framework. No terms | Fidelity | **Procedure and measures:** Mentioned but not included | Not reported |
|  |  | Engagement | **Procedure and measures:** Attendance  Analysis method: Descriptive statistics/percentages | Attendance: Median of 6 group sessions, Median of 8 total sessions. Attendance at group sessions = low: 60% at first session to 20% last session |
| **Olsen et al (2012) ^45^** | No framework. ‘Treatment integrity’ | Fidelity | **Procedure and measures:** 25% of MINT sessions were recorded and rated for treatment integrity by two independent raters using the Behaviour Change Counselling Index (BECCI; Lane et al, 2005). The nurses involved each recorded a subset of their sessions. Each nurse recorded one or two sessions when they had access to the recorder. Measured on a scale of 0-4. Higher scores = greater demonstration of skills. Checks conducted at end of intervention phase.  **Analysis method:** Consistency between raters for the treatment integrity checks was conducted using intra-class correlation coefficients (ICC) for the BECCI rating. Descriptive statistics | BECCI score: between 3.7 and 3.81 out of four. Delivering intervention to a great extent. Inter rater consistency for these scores was excellent (Session one ICC=0.87; Session two ICC=0.99; Session three ICC=0.99). |
|  |  | Engagement | Not measured | Not measured |
| **Osborn et al (2010) ^46^** | No framework used. No terminology/definitions | Fidelity | Not measured | Not measured |
|  |  | Engagement | **Procedure and measures:** Participants asked if they attended the optional diabetes support group. Response options were never, less than 3 months ago, 4 to 6 months ago, 7 to 9 months ago, 10 to 12 months ago, 1 to 2 years ago, and less than 2 years ago  **Analysis method**: Descriptive statistics | Participation in the diabetes support education group: Never 13 (27%), <3 months ago 12 (25%), 4-6 months ago 6 13%), 7-9 months ago 3(6%), 10-12 months ago 4(8%), 1-2 years ago 7(15%), > 2 years ago 3(6%). Control group never 7(16%), <3 months ago 9(21%), 4-6 months ago 9(21%), 7-9 months ago 2(5%), 10-12 months ago 3(7%), 1-2 years ago 7(16%), > 2 years ago 6(14%), |
| **Pettman et al (2008) ^47^** | No framework. Compliance (attendance) | Fidelity | Not measured | Not measured |
|  |  | Engagement | **Procedure and measures:** Compliance with intervention assessed using self-report attendance records and weekly food and PA logs. Weekly attendance recorded each individual for information and PA sessions. If absent, rang to find out why**. S**ubjective assessment was made by two research staff (one actively engaged, one no direct contact) to judge accuracy of records A score was allocated by mutual agreement between the two research staff, of 0 = effectively non-compliant; 0.5 = uncertain or partly-compliant (e.g. not attending exercise sessions, but reported doing additional PA outside of sessions); or 1.0 = appears compliant.  **Analysis method:** Percentage | Attendance: Info session (77%), exercise session (66%), logs (poorly maintained, 1/3 not completing or returning weekly records, 1/3 not completed fully or accurately.). Average = 0.51 (part compliant). |
| **Pill et al (1998) ^48^** | No framework. ‘Clinician competence’, ‘Use of the intervention’ | Fidelity | **Procedure and measures:** Audiotaped consultations analysed to assess clinician competence. Clinicians asked to submit recordings which demonstrated use of the method and were a fair reflection and typical interview. Tapes numbered, transcribed and coded blind using coding framework (which had been refined over several months to ensure reliability/validity).  **Analysis method:** Comparison of audiotaped consultations across groups used the individual consultation as the unit of analysis, where a chi square test was used to compare whether or not the desired behaviour was significantly more likely to be found in the intervention group consultations. | Consultation tapes were provided by all but one of the 29 practices, 51 from 26 experimental group clinicians and 46 from 24 control clinicians. Nurses produced more tapes than doctors (68 vs 29). Nurses covered more topics in the consultation 4.22 vs 2.48 p <0.000) and more often mentioned diet p=0.006 and alcohol use p=0.000, smoking p=0.015, and weight p=0.016)  Components from the 68 nurse tapes (36 intervention, 32 control): Patient decides topic to discuss: 83 vs 72, Patient affirms current behaviour: 100 vs 81 (p=0.006), Patient initiates discussion of change: 50 vs 25 (p=0.03), Any target set 58 vs 41 (NS), Patient sets target 36 vs 28 (NS), Patient takes lead in target setting 28 vs 22 (NS) |
|  |  | Engagement | **Procedure and measures:** Use of the intervention was assessed by telephone interview at the end of the study - how much they were using it in practice  **Analysis method:** Percentages | 71% of clinicians used visual aids frequently, 22% occasionally. Nurses = more attendance (therefore engagement). 2.5 years after the study - 2/3 of the clinicians (22/32) were rated as having a moderate to good understanding of the method and principles. Only six (19%) reported that they were regularly putting it into practice. Relationship between understanding of method and implementation 30 months after recruitment (intervention only). Poor implementation and poor understanding 10, Poor implementation moderate understanding 8, Poor implementation good understand 5, Moderate implementation good understanding 3, Good and good 6 |
| **Roy-Byrnes et al (2010) ^49^** | No framework. ‘Quality of care’ | Fidelity | **Procedure and measures:** Self-report measures of dose, adherence, number and consistency of CBT elements occurring in sessions. For patients in intervention group more detailed information on number and type of sessions extracted from web-based management system.  **Analysis method**: Descriptive statistics | At both 6-month (54.8%;95% confidence interval [CI], 51.0%-58.7%; vs 9.98%; 95% CI, 6.08%-13.88%) and 12-month (21.6%; 95% CI, 18.2%-25.1%; vs 9.31%; 95% CI,5.83%-12.79%) assessments, significantly more patients in the intervention group received psychotherapy with at least 3 of 6 CBT elements (e.g., exposure, relaxation, cognitive restructuring, homework) usually or always delivered. |
|  |  | Engagement | Not measured | Not measured |
| **Saunders et al (2014) ^50^** | Possible frameworks: Baranowski & Stables, 2000; Linnan and Steckler, 2000. Implementation fidelity (extent to which church committees made changes in the environment (Wilcox et al, 2010) | Fidelity | **Procedure and measures:** Participant self-report and provider self-report. Congregant survey: implementation variables for healthy eating and physical activity. ‘‘Getting the message out’’ (healthy eating) assessed by three items; ‘‘providing opportunities’’ by one item; and ‘‘pastor support’’ by one item. ‘‘Getting the message out’’ (physical activity) was assessed by three items; ‘‘providing opportunities’’ by three items; and ‘‘pastor support’’ by two items. All items were rated on four-point scales and church-level means were calculated to reflect level of implementation (higher score = greater implementation).  **Interviewed health directors, pastors and cooks after intervention to assess organisational policies, practices and guidelines in church.** Each item (healthy eating and physical activity) coded yes (1) or no (0) and mean score for each calculated. Lower scores, less implementation. Rarely = 1, Sometimes = 2, Often = 3, Most/all of the time =4  **Analysis method:** descriptive statistics, mediation and ANCOVA | 1. Physical activity: Getting the message out: Intervention: Pre 2.09 (0.53) vs post 2.34 (0.52). Control: Pre 2.23(0.51)) vs post 2.2690.48). Opportunities: Intervention: pre 1.44(0.25) vs post 1.89 (0.58). Control: pre 1.44(0.21) vs post 11.42(0.23). Pastor support: Intervention: pre: 1.67 (0.34) vs post (1.97(0.47). Control: pre: 1.84(0.35) vs post (1.77(0.30). PA policy: Intervention: pre NA post 0.31(0.45). Social support: Intervention: Pre 2.53(0.30) vs post 2.7(0.31). Control: Pre 2.61(0.21) vs post 2.66 (0.32). Self-efficacy: Intervention: pre 2.70(0.24) vs post 2.64 (0.28). Control: pre 2.76(0.24) vs post (2.70 (0.24) 2. Healthy eating: Getting the message out: Intervention: Pre 3.12(0.51) vs poster2.28 (0.54). Control: Pre 2.11 (0.49) vs 2.15 (0.42). Opportunities Intervention: pre 2.87 (0.39) vs post (3.09 (0.43). Control: pre 2.94 (0.32) vs post (3.04 (0.32). Pastor support Intervention: (2.19 0.55) vs 2.55 (0.60). Control: 2.30 (0.38) vs post 2.36 (0.39). PA policy Intervention: NA vs 0.80 (0.27). Control: NA vs 00.30 (0.30). Social support: Intervention: 2.46(0.36) vs 2.64 (0.37). Control: 2.55(0.23) vs 2.64 (0.32). Self-efficacy Intervention: 3.12 (0.16) vs 3.14 (0.24). Control: 3.10 (0.20) vs 3.16 (0.21) |
|  |  | Engagement | Not measured | Not measured |
| **Skidmore et al (2014) ^51^** | No framework. Participants’ comprehension and engagement. Fidelity. | Fidelity | **Procedure and measures:**  All sessions videotaped and 20% from each group randomly selected and rated for fidelity (intervention and control group) against manual using checklists. Checklists developed and validated. Examined treatment integrity (adherence to specified principles, competence in execution, measured by independent raters using yes/no specified principles and ‘inadequate, adequate or exceptional’ competence) and differentiation (raters assessed adherence to determine degree to which it was adhered to and how much the control session did not include elements of intervention. Conditions considered different if adherence ratings were significantly higher for strategy training than attention control.  **Analysis method:** Descriptive statistics and inferential stats not specified | Strategy training: 94% manualized procedures adhered to (85/90). Attention control: 100% manualized procedure adherence. Intervention Therapist demonstrated acceptable/exceptional competence 100% of procedures. Attention Control therapist demonstrated acceptable/exceptional for 99% procedures. The strategy training therapist demonstrated acceptable or exceptional competence for 100% of the completed procedures. Sampled strategy training sessions adhered to 94% of manualized procedures on the strategy training protocol (85 out of 90), Sampled attention control sessions did not contain any of the manualized procedures on the strategy training protocol, indicating good treatment differentiation. Dose: Amount of intervention sessions: M 11.2 vs control 9.5 (T8=-0.53, p=0.61). Duration of sessions: (strategy training M = 37.7 vs 36.8 control (T8=0.19, p=0.86) |
|  |  | Engagement | **Procedure and measures:** Rated participants understanding using a 3 point scale (1-minimal understanding, 2-some understanding, 3-good understanding). Rated participants’ engagement using the Pittsburgh Rehabilitation Participation Scale, a 6 point valid and reliable scale assessing effort and motivation (1-no engagement, 6-excellent engagement). Both measures scored during each session by research therapist.  **Analysis method:** Mean understanding used in analysis. T tests. | Understanding of information: Mean = 2.2 intervention vs 2,6 control (T8=0.84, p=0.43). Participant engagement: strategy training M=4.3 intervention vs 4.0 control (T8=0.31, p=0.77). Strategy training completed 96% of sessions (48/50), attention control 92% of sessions (46/50) |
| **Slade et al (2015) ^52^** | No framework. Attendance and engagement | Fidelity | Not measured | Not measured |
|  |  | Engagement | **Procedure and measures:**  The recovery practice scale used to assess self-rated skills, behavioural intent and behaviour in relation to achieving goals and partnership relationships (15 items. 0-310. High = desirable score). The Participation Scale used to rate attendance and engagement with the personal recovery training, coaching training and team reflection sessions (3 items. Very low to very high. High = desirable score). The Participation scale and recovery practice scale = a non-standardised measures. £10 given to patients after attending assessment and entered into £50 prize draw. Same staff member collected staff data where possible. Data entry protocol followed for consistency and data validation rules applied to reduce transcription errors. All missing data checked manually. Random 20% of RPS checked against paper copies.  **Analysis method:** Not reported – descriptive statistics | Self-rated adherence (average cluster size 9, range 4–16). RPS: Intervention - Skills: 2.79 (0.64). Behavioural intent: 1.66 (0.34), Behaviour 1.78) (0.78). Control - Skills: 2.73 (0.66), behavioural intent 1.68, 09.37), behaviour 1.74 (0.77). Briefing sessions (14): Attendance = 0-25 patients and from 50% to 80% of staff per team. 41/42 personal recovery training sessions: median attendance = 14.4 (range 8-24) session 1, 13.1 (4-21) session 2, 10.4 (6-15) session 3. 42 coaching session run – 14.7 (12-12) team members session 1, 12.0 (7-19 session 2, 11.3 5-14 session 3. Cannot quantify how many staff attended training. 12/36 team reflection groups. Mean attendance 10.0 (5-21). No records kept of reflection groups. |
| **Smith et al (1997) ^53^** | No framework. Treatment adherence (attendance, returning diaries) | Fidelity | Not measured | Not measured |
|  |  | Engagement | **Procedure and measures:** Behavioural measures of adherence: attendance at group meetings, number of diaries turned in, number of days calories were recorded, exercise frequency and number of day’s home blood glucose was monitored. Intervention and control groups.  **Analysis method:** Kruskall Wallis test, analysis of covariance and descriptive statistics | Sessions attended Motivation: 13.3 +- 2.0 vs Standard 8.9 +-2.0 (P=0.01*), Food diaries submitted: Motivation: 15.2 +- 1.8, vs standard: 10.1 +- 2.6 (p=0.01*). Self-monitored blood glucose: Motivational: 46.0 +- 16.1 vs standard: 32.2 +- 10.2 (p=0.05*), Reported exercise Motivational: 35.2 +- 13.2 vs standard: 23.7 +-11.6 (p=0.07*), Recorded calories Motivational: 76.8 +- 15.2 vs standard: 55.7 +- 24.7 (p=0.07*) |
| **Smith et al (2010) ^54^** | No framework/definitions | Fidelity | **Procedure and measures:** Mentions that all care and assessments carried out in keeping with protocol, but not included | Not reported |
|  |  | Engagement | **Procedure and measures:** Monitoring of session attendance  **Analysis method:** Descriptive statistics | 78% attended at least once (1169) – 44% 656 participants completed treatment, 13% transfer of cares, 21% disengaged. 21% people ‘disengaged’ from treatment after being seen at least once. The mean number of contacts for people completing treatment was 5.0 (median 5), and for those disengaging from treatment was 4.1 (median 4). Total mean contact time was 151 min for patients completing treatment (median 135 min), and mean contact time for people who disengaged was 95min (median 80). Response in the treatment complete group was maximal at four to five contacts. |
| **Stanley et al (2013) ^55^** | No framework. Treatment characteristics/adherence | Fidelity | **Procedure and measures:** All sessions audiotaped, and a random 20% were reviewed by an independent treatment integrity rater (did not provide clinical care). Scored: adherence (0 [no adherence] to 8 [optimal adherence]) and competence (0 [no competency] to 8 [excellent competency]).  **Analysis method:** Not reported – percentages | Adequate adherence (5.6 [SD = 1.5]) and competency (5.4 [SD = 1.08]).  Thirteen patients (92.9%) learned behavioural activation, nine (64.3%) learned coping self-statements, and four (28.6%) learned sleep-management skills. |
|  |  | Engagement | **Procedure and measures:** Collateral questionnaires. 1 (dissatisfied to 4 very satisfied – via telephone  **Analysis method:** Not reported – descriptive statistics | Patients completed an average of 3.5 (SD = 2.15) homework exercises per week and spent an average of 81.3 hours (SD = 63.19 hours) per week with the collateral. Four patient-collateral dyads (25%) received handouts to address communication, stress reduction for collaterals, and/or dementia education. Between months 3 and 6, dyads received an average of 5.4 (SD = 3.16) of a possible eight telephone booster calls (66%). 81% collaterals reported using at least one program skill (58% breathing, 50% behavioural activation, 41% calming thoughts) |
| **Suzuki et al (2012) ^56^** | No framework/definitions | Fidelity | Not measured | Not measured |
|  |  | Engagement | **Procedure and measures:** Attendance at intervention sessions  **Analysis method:** descriptive statistics | The mean adherence to the exercise program was 79.2%. 17 subjects (68.0%) more than 80% adherence. |
| **Thyrian et al (2007) ^57^** | No framework/ MI treatment adherence | Fidelity | **Procedure and measures:** Sessions audio-recorded and fidelity was assessed within a single review of a random 20 minute segment of the sessions using the Motivational interviewing treatment integrity (MIT) code - a brief behavioural coding system to measure treatment fidelity for MI. The MITI assesses interviewer behaviour (global ratings and seven behaviour accounts). Global ratings (1=low, 7=high). Behaviour counts do not require a rating but counting of verbal behaviour.  **Analysis method:** Not reported – percentages | Good adherence 66 (48%). Low to moderate adherence 71 (52%) |
|  |  | Engagement | **Not measured** | **Not measured** |
| **Thyrian et al (2010) ^58^** | No framework. Treatment fidelity for MI | Fidelity | **Procedure and measures:** Sessions audio-recorded and fidelity rated using a single review of a random 20 minutes of therapy session using the Motivational interviewing treatment integrity (MIT) code - a brief behavioural coding system to measure treatment fidelity for MI. The MITI assesses interviewer behaviour (global ratings and seven behaviour accounts). Global ratings (1=low, 7=high). Behaviour counts do not require a rating but counting of verbal behaviour. Two researchers (who received supervision regularly) coded every third tape together to assess reliability. Inter rater reliability = fair (r=0.45 for empathy and -.43 for spirit. For behavioural counts – poor to excellent (r=0.25, MI non adherence 4=0.47, complex reflections 0.53, simple reflections 0.58, mi adherent 0.80, giving info r=0.90. Poor interrater reliability for non-adherence to MI may be due to the small range of counts (m=0.84**).** Out of 299 counselling sessions, 161 (54%) were taped, and 84 counselling sessions with current smokers were used in the analysis.  **Analysis method:** Not reported – percentages | Good MI adherence: Total 27 (40%). Low to moderate 40 (60%) |
|  |  | Engagement | Not measured | Not measured |
| **Tomasone et al (2014) ^59^** | No framework. Intervention delivery components | Fidelity | **Procedure and measures:** Presenter checklist completed after each seminar. Seven intervention components (number of attendees, duration, parasport athlete present, parasport equipment available, educational resources attributed, inclusion of audio-visual component, and partner with community organisations. Measured with yes/no. As a reliability check the first author attended and completed a presenter checklist for two CMCL seminars delivered by two different presenters. Checklist items included in the current study (n=7) agreement between the researcher and presenters were high (86% and 100%)  **Analysis method:** Not reported – descriptive statistics | Completed for 14/15 seminars delivered. Number of attendees presents 8–77. Duration (minutes) 60–120. Parasport athlete present 85 yes 7 no. Parasport equipment available for viewing and use 42 yes 50 no. Educational resources about LTPA for people with a physical disability distributed 87 yes 5 no. Inclusion of audio-visual component (e.g., photos, videos) not part of standard CMCL curriculum 14 yes 78 no. Partner with community organization 21 yes 71 no |
|  |  | Engagement | Not measured | Not measured |
| **Van de Glind et al (2012) ^60^** | Hasson et al (2010) proposed a framework for evaluation of implementation – based on Carroll et al (2007). Program adherence | Fidelity | **Procedure and measures:** Quality of the delivery of implementation strategies (implementation fidelity): registration by the researchers and checking to what extent strategies were carried out as planned in interviews with nurses and managers. *Frequency and duration* of counseling sessions registered by nurses on an evaluation form. The *content* of the counseling was categorized by analyzing patient files (patient files reviewed to see if anamneses carried out (yes/no), how many info leaflets handed out, goals formulated and written down (at least one), patients motivation assessed and registered (at least one) (yes/no), self-efficacy assessed and registered (at least one) (yes/no). barriers and facilitators discussed and written down (at least one) (yes/no). Development of measure – literature, linked the factors by two researchers, considered theories, presented a timetable of what when and who should do what, decided strategies that were compulsory/desired and sent this for feedback to key people. To create a final version  **Analysis method:** Coverage score computed and written down – percentage of components delivered as planned. 80-100% = high. 50-80% moderate. Less than 50% = low. | Moderate to good adherence to protocol (65%–90%). Perceived implementation success (1-10) (interviews with nurses). Case 1: 5, Case 2 4.9, Case 3 6.0, Case 4 5.3, Case 5 7.0. Frequency: Amount of consultations: Case 1: 100%, 2.7, Case 2: 100% 2.5, Case 3: 100% 2.3, Case 3: 100% 1.4, Case 5: 1.4. Duration: Mean in minutes: Case 1: 100% 159, Case 2: 100% 108, Case 3: 100% 124, Case 4: 100% 98, Case 5 100% 60. Content: Anamneses: Case 1: 100%, Case 2 100%, Case 3 100%, Case 4 100%, Case 5 100%, Information leaflet: Case 1: 80%, Case 2 100%, Case 3 100%, Case 4 100%, Case 5 100%, Goal setting: Case 1: 57%, Case 2 95%, Case 3 71%, Case 4 38%, Case 5 60%, Motivation assessment: Case 1: 43%, Case 2 83%, Case 3 86%, Case 4 25%, Case 5 40 %, Self-efficacy assessment: Case 1: 29%, Case 2 83%, Case 3 86%, Case 4 25%, Case 5 40%, Barriers and facilitators assessment: Case 1: 14%, Case 2 58%, Case 3 86%, Case 4 38%, Case 5 40-%, Coverage % elements delivered per patient: Case 1: 65%, Case 2 90%, Case 3 91%, Case 4 66%, Case 5 73% |
|  |  | Engagement | Not measured | Not measured |
| **Wallace et al (1998) ^61^** | No framework/definition | Fidelity | Not measured | Not measured |
|  |  | Engagement | **Procedure and measures:** Attendance records and nurse follow up phone interviews (reading of tip sheets/use/meeting goals)  **Analysis method:** Percentages | Attendance at classes: 90%+. After 6m trial, 51% controls joined intervention, half intervention continued to attend. 92% read nutritional tip sheets, 82% reported they were useful, 90% reported meeting some or all of nutritional goals. |
| **Weinberger et al (2002) ^62^** | No framework. ‘dose of the intervention’ | Fidelity | **Procedure and measures:** Log file captured each time the intervention pharmacists accessed a patient’s record from the study computer or documented their actions on the computer. Frequency with which pharmacists documented actions was used to estimate the dose of the intervention  **Analysis method:** Descriptive statistics and odd ratios**.** Repeated measures approach **–** compound symmetry variance covariance structure? | Number of visits: program (mean 19.4 (16.8) visits and COPD (22.4 (17.4) visits). Accessed data: asthma 10.3 (7.5 visits) COPD 11.8 (10.5) visits. Documented actions: (asthma 6.2 (5.8 visits) COPD 6.2 (7 visits). When pharmacists documented more care actions patients exhibited less noncompliance with breathing medication (OR 0.96 CI 0.92-0.99). and hospital visits (OR 1.06, 1.04-1.07, p<0.001) |
|  |  | Engagement | **Not measured** | **Not measured** |
| **Welch et al (2011) ^63^** | No framework. Treatment fidelity | Fidelity | **Procedure and measures:** Sessions audio-recorded, random sample of 24 sessions chosen midpoint of intervention phase assessed by independent researcher using Motivational Interviewing skills code (1-7 Likert scale – frequency of educator behaviours, behaviour count and count of client responses)**.** Treatment fidelity in training phase assessed using Motivational Interviewing Treatment integrity code (MITI) v2.  **Analysis method:** Descriptive and t test | MEAN MI spirit rating: ICC: 0.77, MI threshold proficiency >5. MI trained educators 4.43, non-mi trained educators 2.65, t= 11.26, p<0.001. Ratio of reflections to questions: ICC=0.65, proficiency 1:1. MI trained educators, 1.92, non-mi trained 0.23, t= -6.59, p<0.001. Percentage of open questions: ICC = 0.93, MI threshold proficiency 50%, MI trained 0.27, non-mi trained 0,06, t=-7.37, p<0.001. Mean count of MI inconsistent responses ICC = 0.75, proficiency n/a, MI trained educators 3.27, non-MI trained 12.19, t=9.38 p<0.001. Mean count of client change talk ICC = 0.66, MI trained 13.88, non-MI trained 9.02, t=-2.93, p=0.004 |
|  |  | Engagement | Not measured | Not measured |
| **West et al (2007) ^64^** | No framework. ‘Fidelity monitoring’ | Fidelity | **Procedure and measures:** Ongoing clinical supervision of MI skills and fidelity were combined in weekly supervision sessions  Randomly selected audio-tapes were reviewed weekly by two clinical psychologists using a standardised coding format modelled on other studies of MI proficiency (assessed overall MI spirit, presence of behaviours, absence of behaviours and proportion counsellor spent talking), using a standardised tool  **Analysis method:** Not reported | Not reported |
|  |  | Engagement | **Procedure and measures:** To determine engagement, number of group sessions attended, number of self-monitoring diaries submitted and average group leader rating of self-monitoring diary quality (3 point scale) were measured  **Analysis method:** Percentages | Attendance at individual MI/AC sessions: 0-6 months 1.9 +- 0.3, 6-12m 2.3 +1.0, Total (5) 4.2 +- 1.2. Attending >80% sessions (%): 81. Group sessions attended (%): 6m: Total: 72, MI 79, Control 71, 12m: 57, 62, 52, 18m: 48, 52, 43. Diaries submitted: 6m 15 +- 8 total, 17 +- 8, MI, 13 +- 8, control, 12m: 7 +9, 9 + 10, 5 + 7, 18m 5+9, 6 + 10, 3 + 7 |
| **Wieland et al (2012) ^65^** | No framework/definitions | Fidelity | Not measured | Not measured |
|  |  | Engagement | **Procedure and measures:** Attendance was charted at each class. Satisfaction with the classes was assessed through seven items from the previously validated Physical Activity Class Satisfaction Questionnaire (Cunningham, 2007) (at end of the intervention).  **Analysis method:** Descriptive statistics | Average attendance (each class) 22.5. 32 women completed evaluation |
| **Windsor et al (2014) ^66^** | No framework. Fidelity | Fidelity | **Procedure and measures:** 7 script procedures documented by a process evaluation model using standardised tele forms. The SCRIPT PII documents the degree to which the seven core procedures were delivered with fidelity. DCC performance data were aggregated to compute an annual RFTS-SCRIPT program implementation index  A PII = 100% would confirm that all clients of all DCCs received all SCRIPT program procedures. A SCRIPT PII 80% = good level of implementation (selected by committee).  **Analysis method:** Percentages | The RFTS-SCRIPT Program Implementation Index (PII) improved from a PII = 65 % in 2006 to PII = 76 % in 2010. 20 % of DCCs had a PII B 50 %, and 25 % of RFTS clients (n = 103) who wanted SCRIPT in 2009–2010 did not receive it. |
|  |  | Engagement | Not measured |  |
